# Cold‐inducible promoter‐driven knockdown of *Brachypodium* antifreeze proteins confers freezing and phytopathogen susceptibility

**DOI:** 10.1002/pld3.449

**Published:** 2022-09-12

**Authors:** Collin L. Juurakko, Melissa Bredow, George C. diCenzo, Virginia K. Walker

**Affiliations:** ^1^ Department of Biology Queen's University Kingston Ontario Canada; ^2^ Present address: Department of Plant Pathology and Microbiology Iowa State University Ames Iowa USA; ^3^ School of Environmental Studies Queen's University Kingston Ontario Canada

**Keywords:** abiotic stress, antifreeze proteins, *Brachypodium distachyon*, freeze tolerance, ice‐recrystallization inhibition, *Pseudomonas syringae*

## Abstract

The model forage crop, *Brachypodium distachyon* , has a cluster of ice recrystallization inhibition (*BdIRI*) genes, which encode antifreeze proteins that function by adsorbing to ice crystals and inhibiting their growth. The genes were targeted for knockdown using a cold‐induced promoter from rice (pr*OsMYB1R35*) to drive miRNA. The transgenic lines showed no apparent pleiotropic developmental defects but had reduced antifreeze activity as assessed by assays for ice‐recrystallization inhibition, thermal hysteresis, electrolyte leakage, and leaf infrared thermography. Strikingly, the number of cold‐acclimated transgenic plants that survived freezing at −8°C was reduced by half or killed entirely, depending on the line, compared with cold‐acclimated wild type plants. In addition, more leaf damage was apparent at subzero temperatures in knockdowns after infection with an ice nucleating pathogen, *Pseudomonas syringae* . Although antifreeze proteins have been studied for almost 60 years, this is the first unequivocal demonstration of their function by knockdown in any organism, and their dual contribution to freeze protection as well as pathogen susceptibility, independent of obvious developmental defects. These proteins are thus of potential interest in a wide range of biotechnological applications from cryopreservation, to frozen product additives, to the engineering of transgenic crops with enhanced pathogen and freezing tolerance.

## Graphical abstract

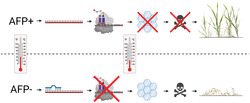

## 1 INTRODUCTION

Increased freezing events and altered freeze–thaw cycles in response to climate change present major challenges to agriculture with single frosts costing billions of dollars (Kreyling, 2019; NOAA, 2021; Sinha & Cherkauer, 2010; Smith et al., 2021; Smith & Katz, 2013; Witney & Arpaia, 1991). In the field, the formation of ice at high sub‐zero temperatures is initiated by ice nucleation active (INA+) bacteria and is a major driver of crop destruction (Snyder & Melo‐Abreu, 2005). Because they cannot escape low temperatures, many temperate climate plants have adopted a freeze tolerant strategy with some producing ice recrystallization inhibition (IRI) or antifreeze proteins (AFPs) to help prevent freeze damage (e.g., Gupta & Deswal, 2014; Juurakko, diCenzo, & Walker, 2021). In contrast, other organisms such as polar fish and temperate arthropods, which can escape low temperatures and find hibernacula, frequently adopt a freeze‐avoidance strategy that can also employ AFPs, in this case to lower the freezing point relative to the melting point, also known as the thermal hysteresis (TH) gap (Bar Dolev et al., 2016; Duman, 2001; Kim et al., 2017). Despite the discovery of these proteins, first recognized almost 5 decades ago in *Tenebrio* beetles (Ramsay, 1964), until recently, there had been no formal evidence of their contribution to low temperature survival using gene deletions or gene expression “knockdowns” in any organism. This changed with the engineering of transgenic grass lines with translational knock down expression of AFPs, resulting in greater freeze susceptibility (Bredow et al., 2016). However, important as these results were, it was worrying that the knockdowns were associated with other phenotypes including stunted growth and almost complete sterility, suggesting to critics that greater freeze susceptibility in these transgenic lines was simply due to unhealthy plants rather than lower AFP activity. To test that possibility, and to verify that AFPs do indeed make a crucial contribution to freeze survival, it was important that new transgenic lines be made that were unencumbered with those detrimental developmental phenotypes.

AFPs or IRI proteins are also known as ice‐binding proteins. The plant freeze‐tolerant overwintering strategy appears to be associated with AFPs that are characterized by low TH activity but a high IRI activity, which keeps ice crystals small even when the temperature fluctuates near 0°C. This is important as ice forms in the apoplast, frequently due to INA+ bacteria, such as *Pseudomonas syringae*, gaining entry through stomatal openings, hydathodes, or wounding sites that themselves may result from surface tissue ice formation (Ashworth & Kieft, 1995; Lindow et al., 1982; Pearce & Fuller, 2001; Wisniewski & Fuller, 1999). Some plant AFPs have even been shown to attenuate the INA+ activity of *P. syringae*, possibly by binding to the bacterial ice nucleating proteins (INPs), resulting in a modest lowering of the freeze temperature (Bredow et al., 2018; Tomalty & Walker, 2014). The apoplast has a lower solute concentration and thus freezes sooner relative to other compartments or specific tissue. Thus, to combat catastrophic freezing, AFPs are produced and secreted to the apoplast (Antikainen & Griffith, 1997; Bredow et al., 2016; Griffith et al., 1992; Hon et al., 1994, 1995; Marentes et al., 1993; ). The property of AFPs to irreversibly adsorb to ice crystals is key to membrane protection and explains why these proteins can be employed in both freeze‐tolerant and freeze‐avoidant strategies. In the absence of other ice‐management mechanisms, uncontrolled ice growth in the apoplast can lead to cellular death by dehydration through exclusion of solutes or the piercing of membranes, thus presenting the primary battleground between AFPs and ice (Lindow et al., 1982; Melo‐Abreu et al., 2016).

*Brachypodium distachyon* (hereinafter *Brachypodium*) contains 7 ice‐recrystallization inhibition genes (*BdIRI1‐7*). The gene translation products are hydrolysed, likely in the apoplast, to generate two independent proteins, a leucine‐rich repeat (LRR) protein and an AFP (Bredow et al., 2016). The *BdIRI* gene sequences are sufficiently similar so that a single artificial miRNA could be designed that attenuated the translation of all 7 corresponding mRNAs with no obvious off‐target binding (Bredow et al., 2016). As noted, previously generated transgenic *Brachypodium* lines bearing the miRNA sequence, driven by the constitutive CaMV 35S promoter, were more susceptible to freeze damage than non‐transgenic controls. Thus, although these experiments clearly connected AFPs with freeze protection, as the lines also showed developmental deficits, we hypothesized that a cold‐induced promoter, perhaps more similar to the native *BdIRI* promoters, would circumvent this problem and allow a fuller characterization of the newly generated AFP knockdown lines. This has now been achieved. We additionally report for the first time, an exploration of *BdIRI* regulation as well as a demonstration that the experimental temporal attenuation of AFP expression is inextricably linked to greater freeze susceptibility including that triggered by infection with the INA+ phytopathogen, *P. syringae*.

## 2 METHODS

### 2.1 Bioinformatic analysis

*BdIRI* gene and protein sequences were retrieved from NCBI using up‐to‐date accessions (December 2020) using BLAST searches with the published proteins (Bredow et al., 2016). The lack of known suitable low‐temperature inducible promoters in *Brachypodium* prompted the selection of the 1961‐bp promoter associated with the rice, *Oryza sativa*, gene *OsMYB1R35*, which is induced in its host plant after cold stress (Li et al., 2017). The sequence was retrieved from the *O. sativa* genome (National Centre for Biotechnology Information or NCBI; accessed October 2017) based on the primer sequences (Li et al., 2017). The 1961‐bp fragment was synthesized by GeneART (Thermo Fisher Scientific, Waltham, MA) with appropriate flanking restriction enzyme recognition sites. Conceptually translated *BdIRI* sequences were aligned and phylogenies prepared using the Clustal Omega Multiple Sequence Alignment tool (https://www.ebi.ac.uk/Tools/msa/clustalo/; Sievers et al., 2011). Inter‐domain hydrolytic cleavage sites in the amino acid sequence corresponding to the *BdIRI*s were predicted using the ExPASy PeptideCutter tool (https://web.expasy.org/peptide_cutter/; Gasteiger et al., 2005). Chromosomal positioning of the *BdIRI* genes was determined using NCBI's genome browser. InterProScan (Version 83.0; https://www.ebi.ac.uk/interpro/) was used for the in silico prediction of the LRR and AFP domains (Blum et al., 2021). The Phyre2 Protein Fold Recognition Server (http://www.sbg.bio.ic.ac.uk/~phyre2/) was used for sequence‐based homologous protein structure predictions (Kelley et al., 2015), and the SignalP 5.0 Server (http://www.cbs.dtu.dk/services/SignalP/) was used to identify putative secretory signal peptides (Armenteros et al., 2019). The DeepLoc 1.0 eukaryotic protein subcellular localization predictor (http://www.cbs.dtu.dk/services/DeepLoc-1.0/) was used to check the subcellular localization of proteins secreted to the apoplast (Armenteros et al., 2017).

### 2.2 Prediction of miRNA targets and analysis of regulatory elements

Identification of post‐transcriptional regulation via endogenous microRNAs was performed on the *BdIRI* mRNA sequences using the psRNATarget miRNA prediction tool (http://plantgrn.noble.org/psRNATarget/; Dai et al., 2018) using data from miRBase (Release 21, June 2014) (Griffiths‐Jones, 2004; Griffiths‐Jones et al., 2006; Griffiths‐Jones et al., 2007; Kozomara et al., 2019; Kozomara & Griffiths‐Jones, 2010; Kozomara & Griffiths‐Jones, 2014).

Sequences upstream to translational start codons were retrieved from NCBI (*B. distachyon* genome assembly v3 2020) in addition to the promoter sequence of p*rOsMYB1R35* (Li et al., 2017). PlantCARE (http://bioinformatics.psb.ugent.be/webtools/plantcare/html/; Lescot et al., 2002) was used for *cis*‐regulatory element prediction and analysis. *Cis*‐regulatory elements specifically related to cold signaling and cold stress were manually annotated for putative promoter sequences of all *BdIRI*s and *OsMYB1R35*, designated as pr*BdIRIs1‐7* and pr*OsMYB1R35*, respectively.

### 2.3 Plasmids construction and *Brachypodium* transformation

The pr*OsMYB1R35* promoter sequence in pUC was transformed into *Escherichia coli* DH5α cells (Thermo Fisher Scientific) and subsequently liberated from purified plasmid using *Bam*HI and *Bgl*II restriction enzymes at the 5′ and 3′ end, respectively. It was then ligated into pCambia1380 (Marker Gene Technologies Inc., Eugene, OR, USA) and transformed into *E. coli* DH5α. A plasmid bearing a sequence corresponding to the artificial miRNA (TAGGTTGAGCGACTCCCACTG; Bredow et al., 2016) with a 5′ *Bgl*II site was amplified by polymerase chain reaction (PCR) and a 3′ *Spe*I restriction site was added. Similarly, the sequence encoding enhanced green fluorescent protein (eGFP; GenBank Accession no. U57607) was PCR‐amplified, and 5′ *Bgl*II and 3′ *Spe*I restriction sites were added. After digestion of the inserts with *Bgl*II and *Spe*I, the amplified products, miRNA and eGFP, were ligated separately to create the plasmids pCambia1380:pr*OsMYB1R35*:miRNA and pCambia1380:pr*OsMYB1R35*:eGFP, respectively. These were independently transformed into *E. coli* DH5α and then confirmed by Sanger sequencing (CHU de Québec‐Université Laval, Quebec City, QC, CA). Subsequentially, the plasmid DNA was transformed into *Agrobacterium tumefaciens* (AGL1, Invitrogen, Carlsbad, CA, USA) (hereinafter, *Agrobacterium*), and sequence confirmed before the cells were used for *Brachypodium* transformation.

Transgenic *Brachypodium* lines were generated using a modified method from Fursova et al. (2012) by using transformed *Agrobacterium* cultures (50 ml), grown in Luria Bertani (LB) broth with 50 mg L^−1^ kanamycin to an OD_600_ of 1, pelleted at 5000 × *g* for 10 min, and washed in equal volumes of 2‐(N‐morpholino)ethanesulfonic acid (MES) infiltration buffer (10‐mM MES, 10‐mM MgCl_2_, pH 5.6). Pellets were resuspended in 50 ml of infiltration buffer containing 50 μM acetosyringone, 0.01% Silwet‐L77 organosilicone surfactant, and leaf extracts containing phenolic metabolites to initiate efficient Ti plasmid transfer made from Australian tobacco, *Nicotiana benthamiana* leaves, rather than *N. tabacum* (Fursova et al., 2012). Tobacco extracts were prepared using ~50 g of leaf tissue from 6‐week‐old plants, cut into 1–3 cm^2^ squares, and incubated in 300 ml of infiltration buffer for 2 h before removing and subsequently filter‐sterilizing the recovered liquid through 0.22‐μm syringe filters (Thermo Fisher Scientific). Acetosyringone and surfactant were added following filter sterilization and used to resuspend the washed *Agrobacterium* pellet. In parallel, mature wild type seeds (50 per trial) were harvested, surface‐sterilized, and trimmed using a sterile scalpel to remove the upper quarter of the seed. The balance of the seeds, with exposed embryos, was immediately added to the now‐primed *Agrobacterium* culture and co‐cultivated for 30 h at 21°C with shaking at 200 rpm, in the dark.

Following co‐cultivation, seeds were washed in infiltration buffer and plated on Linsmaier and Skoog (LS) agar (Phytotech Labs, Lenexa, KS, USA) containing 225 mg L^−1^ timentin. Plates were sealed and put at *Brachypodium* standard growth conditions (see below). One week after successful germination, surviving T_0_ generation seedlings were sown to soil with DNA then extracted using Monarch DNA extraction kits (New England Biolabs, Ipswich, MA, USA) and sequence confirmed (Université Laval, Quebec City, QC, Canada). After T_0_ plants were brought to senescence, seeds were counted, and 10 seeds per line were sterilized and germinated on LS agar with hygromycin. These were then sown in soil and brought to senescence representing the T_1_ generation. After collection, seeds (24 per line) were sterilized and germinated on LS agar without antibiotics. These T_2_ generation plants were used for rapid genotyping (see below) to determine homozygosity for the T_3_ generation.

### 2.4 Genotyping *Brachypodium* transgenic lines

Genotyping was done using a method modified from Ben‐Amar et al. (2017). Briefly, seeds (24 per line) were sterilized, germinated, and sown in soil. Leaf tips (~2 mm long) were cut at 2 weeks and placed in 96 well PCR plates containing 50 μl of TE buffer at pH 8.0. After grinding the leaf tissue, the plates were incubated at 60°C for 10 min and vortexed. Extracted samples (2 μl) from each line were combined and vortexed, and 1 μl was used in a PCR screen for the insert. Individual sample screening was conducted when pooled extracts showed an amplified DNA band of an appropriate size. A line was considered homozygous if every individual showed the same band. A reference gene, S‐adenosylmethionine decarboxylase (*SAMDC*), was used as a PCR control.

### 2.5 Western blot analysis

Western blot analysis was conducted on leaf samples from 3‐week‐old wild type (ecotype Bd21; RIKEN, Wakō, Japan) plants or transgenic plants maintained under standard conditions (non‐acclimated; NA) or transferred to 4°C for 48 h (cold‐acclimated; CA, see details below). Seeds from wild type or transgenic pr*OsMYB1R35*:eGFP plants were surface sterilized and then germinated on 50 mg mL^−1^ hygromycin B selection medium (BioShop Canada Inc., Burlington, ON, Canada). After sowing in potting soil, they were grown for 3 weeks before harvesting 200 mg of leaf tissue from each plant. The tissue was frozen with liquid nitrogen, ground using a sterile micro pestle, suspended in extraction buffer (5‐mM DTT, 1% Sigma P9599 protease inhibitor, 0.1% Igepal, 2‐mM NaF, 1.5‐mM activated Na_3_VO_4_, 0.5‐M Tris–HCl pH 7.5, 10% glycerol, 0.15‐M NaCl) and then shaken at 150 rpm at 4°C for 4 h. Following centrifugation (13,000 × *g* at 4°C for 30 min), protein concentration in the supernatant was estimated (Bradford reagent, Thermo Fisher Scientific) and then diluted so that all samples were equivalent. Each sample (100 μl) was then denatured by the addition of 50 μl of Laemmli Sample Buffer (45% glycerol, 10% SDS, 0.5‐M Tris pH 6.8, 0.045% w/v bromophenol blue, 0.006% 1 M DTT) and boiled for 5 min. Samples were electrophoresed using a semi‐dry transfer apparatus (Bio‐Rad Laboratories, Hercules, CA, USA) following the manufacturer's recommended protocols. The membrane was blocked for 1 h using a 5% (w/v) skim milk powder in Tris‐buffered saline with 0.1% Tween® 20 detergent (TBST) while shaking at room temperature.

GFP was detected on the membranes using anti‐GFP mouse‐IgG monoclonal (clones 7.1 and 13.1) antibody (Roche, Basel, Switzerland) in a 1/500 dilution in 5% (w/v) skim milk powder TBST solution with gentle shaking overnight at 4°C in the dark. The secondary antibody, anti‐mouse IgG peroxidase‐conjugated (Sigma‐Aldrich, St. Louis, MO, USA), was used at 1/4000 dilution in the skim milk‐TBST solution with shaking at room temperature for 1 h. Coomassie brilliant blue stained ribulose‐1,5‐bisphosphate carboxylase‐oxygenase (better known as RuBisCO) large chain (RbcL) was used as a 55‐kDa loading control. Blots were washed with TBST buffer three times for 10 min, inserted between acetate transparency sheets and imaged on a ChemiDoc Touch Imaging System (Bio‐Rad Laboratories) using Immobilon western chemiluminescent HRP substrate (MilliporeSigma, Boston, MA, USA). Western blot analysis was done using Image Lab Software (Bio‐Rad Laboratories). Purified recombinant eGFP was used as a positive control, and western blots were repeated in triplicate.

### 2.6 Plant material and growth conditions

*Brachypodium* Bd21 seeds were soaked in sterile water for 1 h, and the lamella, awn, and any remaining appendages still attached to the harvested floret were removed. The seeds were briefly washed in a 40% bleach, 0.04% w/v Silwet‐L77 solution followed by a 70% ethanol rinse, and 4 rinses in sterile water before being dried on filter paper soaked in 100% ethanol. All open seed work was done in a UV sterilized laminar flow hood. After seed transfer to LS agar plates using sterilized forceps, the plates were subsequently sealed, wrapped in foil, and kept at 4°C for 4 days. The plates were then moved to a climate‐controlled growth chamber (Conviron CMP4030, Controlled Environments Limited, Winnipeg, MB, Canada) at standard *Brachypodium* growth conditions of 70% relative humidity and 24 h cycles of 20 h light (~150 μmol m^−2^ s^−1^) at 24°C followed by 4 h with no light at 18°C. After 1 week, seeds were transplanted to potting soil Sunshine® Mix #1 (Sun Gro® Horticulture, Agawam, MA, USA) and fertilized bi‐weekly using 10‐30‐20 Plant‐Prod MJ Bloom (Master Plant‐Prod, Brampton, ON, Canada). Prior to assay, CA plants were moved to a separate chamber (Econair GC‐20, Ecological Chambers Inc., Winnipeg, MB, Canada) maintained at 4°C where they were subjected to a shortened day cycle of 6 h of light (~150 μmol m^−2^ s^−1^) and 20 h of darkness for 48 h. NA plants remained at standard conditions.

### 2.7 Crude lysate and apoplast extract preparations

AFP activity was assayed in extracts prepared as described (Bredow et al., 2016). Leaf tissue (50 mg) from the 3‐week‐old plants was frozen with liquid nitrogen and ground to a powder and suspended in 400 μl of NPE buffer (25‐mM Tris, 10‐mM NaCl, pH 7.5, EDTA‐free protease inhibitor tablets), with the slurry shaken for 4 h at 4°C in the dark on a GyroMini nutating mixer (Labnet International, Edison, NJ, USA). Samples were centrifuged at 13,000 × *g* for 5 min, placed at 4°C for 5 min, and recentrifuged with the supernatant transferred to 1.5‐ml tubes and centrifuged again (13,000 × *g* for 5 min) before returning to 4°C. Protein concentration was estimated using a Nanodrop One (Thermo Fisher Scientific; using a mass extinction coefficient [ɛ_1%_] of 10 at 280 nm for 10 mg mL^−1^ with a baseline correction at 370 nm, as recommended by the manufacturer). Readings were performed in triplicate for each sample, with samples routinely diluted to 1 mg ml^−1^ unless stated otherwise.

Apoplast extracts were prepared as previously described (Pogorelko et al., 2011) with minor modifications. Briefly, 500 mg of leaf tissue from 3‐week‐old NA and CA wild type and knockdown lines was collected. Leaf tissue was aseptically sliced into 1‐cm segments, placed vertically into a 10‐ml syringe, and the tip sealed with parafilm. Extraction buffer (5 ml of 25‐mM Tris–HCl, 50‐mM EDTA, 150‐mM MgCl_2_, pH 7.4) was added, and the syringe was placed under vacuum for 1 min, four separate times, until the leaves were fully infiltrated when the tissue was transferred to a 3‐ml syringe, the excess buffer drained, and the barrel was placed into a 15‐ml conical tube and centrifuged at 1000 × *g* for 10 min at 4°C. Protein concentration of the recovered apoplast fluid was estimated using a Synergy H1 microplate reader (BioTek Instruments, Inc., Winooski, VT, USA) with a Take3 Micro‐Volume Plate (BioTek Instruments) at A_280_, as described for the lysates. Samples were diluted appropriately as described prior to assaying. All work was carried out at 4°C.

### 2.8 AFP assays

Assays for AFPs assess different properties of these proteins. Indeed, there are no clear correlations between levels of AFP activity as measured by TH, IRI, ice crystal morphology, and presumably electrolyte leakage for different AFPs (e.g., Gruneberg et al., 2021). Thus, to determine the impact of the translational inhibition of *BdIRI* transcripts, a variety of assays were employed. IRI activity was assessed using a modified “splat” assay as previously described (Bredow et al., 2017). Briefly, samples (10 μl) were pipetted 1 m above a glass cover slip, equilibrated on an aluminum block chilled with dry ice, to ensure the formation of a thin layer of small ice crystals prior to transfer into a hexane‐containing bath at a −6°C annealing temperature. Images were captured through cross‐polarizing films at 10× magnification, immediately after transfer to the bath and again after 18 h. Negative (buffer) controls as well as positive controls consisting of ice‐purified perennial rye grass, *Lolium perenne* AFP (Bredow et al., 2016; Bredow & Walker, 2017), were routinely employed, and lysates and apoplast extracts were subjected to a standardized dilution series with assays done a minimum of three times for all samples.

TH assays were as described (Bredow et al., 2020) but with 200 mg of leaf tissue and 800 μl of buffer. Amicon Ultra‐0.5 micro‐concentrators (Millipore) were used to concentrate supernatants 4‐fold after centrifugation (13,000 × *g* for 10 min). TH was determined on a nanoliter osmometer (Middleton et al., 2014). Ice crystal morphologies can also be used to assess AFP activity, and these were recorded during the TH assays with images captured using a microscope video camera at 50× magnification and in triplicate.

### 2.9 Electrolyte leakage, infrared thermography, whole plant freezing assays, and infections

Electrolyte leakage assays were as described (Bredow et al., 2016). Briefly, leaf tips (~4 cm long) were excised from 3‐week‐old plants and individually placed into 100 μl of deionized water. Control tubes were kept at 4°C in the dark, but each companion set of tubes was placed in a programmable circulating ethylene glycol temperature‐controlled bath set at 0°C. The temperature was ramped down from 0°C to −1°C, over 30 min, when a single ice chip was added to initiate ice crystal growth, and the temperature was lowered 1°C every 15 min until the final freezing temperature of −10°C was reached. Both sets of tubes were then left overnight at 4°C in the dark. Subsequently, the tube contents were transferred to 50‐ml centrifuge tubes containing 25 ml of deionized water and shaken horizontally on a G2 Gyrotory Shaker (New Brunswick Scientific, Edison, NJ, USA) at 150 rpm for 18 h in the dark at room temperature. Initial conductivity (C_i_) was measured using an Oakton CON 510 conductivity meter (OAKTON Instruments, Vernon Hills, IL, USA) prior to autoclaving the samples for 45 min. After cooling overnight, the final conductivity (C_f_) was measured with percent electrolyte leakage calculated as (100 × C_i_/C_f_). The assay was performed in triplicate using leaves from 10 individual plants per line and condition.

To visualize ice propagation in leaves and the influence of AFPs, infrared thermography was used to detect the emitted infrared energy. Typically, plant emissivity ratings are in the range of 0.98 (Chen, 2015; López et al., 2012), but to enhance the contrast, aluminum foil (emissivity of 0.05) was used as a background (Qin et al., 2017). The FLiR One Pro – iOS (FLIR Systems, Wilsonville, OR, USA) with Vernier Thermal Analysis Plus application (Apple App Store) (Vernier Software and Technology, Beaverton, OR, USA) was used to capture thermography data. *Brachypodium* leaves of equal length (~2.5 cm) were freshly excised from CA 3‐week‐old plants and placed on a stage lined with foil touching the surface of a circulating ethylene glycol bath set at 1°C. Leaf tissue was annealed for 30 min before distilled water (10 μl) was pipetted onto the wounded end of the leaf and sterile ice chips of equal size were added to each sample to initiate nucleation. The temperature was then lowered to −10°C at 0.01°C s^−1^. Temperature measurement points were set with the software for each leaf ~1 cm from the wound and recorded as freezing progressed. Data were analyzed using Logger Pro (version 3.14) (Vernier Software and Technology). The assays were performed in triplicate.

Assessment for whole plant freeze survival was modified from previous methods (Bredow et al., 2016; Colton‐Gagnon et al., 2014; Mayer et al., 2020). *Brachypodium* seeds (10 per line and condition) were sterilized and sown in 10 × 15 × 7.6‐cm pots, as described in plant growth conditions. Seeds were stratified in darkness at 4°C for 4 days and then seedlings were grown at standard, previously described conditions for 2 weeks. The pots were then transferred to another climate‐controlled chamber (Econair GC‐20; Ecological Chambers Inc.) and exposed to −1°C for 12 h before the lights were turned off and temperature was ramped down at a rate of 1°C per h until −8°C and after which the temperature was returned to 4°C and the lighting resumed (~150 μmol m^−2^ s^−1^) for 24 h. After recovery, plants were returned to standard conditions for 2 weeks, survival was recorded, and images captured. Survival assays were repeated in triplicate.

Infections at high subzero temperatures were assayed using liquid cultures of *P. syringae* pv. *syringae* B728A, a pathovar with ice nucleation activity (Feil et al., 2005). The bacteria were cultured while shaking at 28°C to OD_600_ = 0.6–1.0 and then shaken at 4°C for 2 days to increase INP production and subsequently resuspended in 10‐mM MgCl_2_ and diluted to an OD_600_ of 0.2, corresponding to ~1 × 10^8^ colony forming units (CFUs) mL^−1^. Simultaneously, 3‐week‐old plants were CA at 4°C for 2 days well apart from the bacterial cultures. Leaves of equal size and length were aseptically removed, and the wound was dipped in the bacterial cultures. Leaves were then incubated at −3°C in an enclosed temperature‐regulated chamber for 12 h, assessed for evidence of disease including water soaking and cell death and then allowed to recover at 4°C in the dark with reassessment at 24 h, 48 h, and 1 week. Plants were grown at standard conditions for 3 weeks with NA and CA plants sprayed with culture as well as cut leaves exposed to wound dipping as described. Whole plants and leaf tissue infections were carried out at −3°C and recovered as described with separate plants and leaf tissue maintained at standard *Brachypodium* conditions following infection as controls. All assays were repeated a minimum of three times.

### 2.10 Statistical analysis

All statistical analyses were performed in R (version 4.1.1) using the package multcomp for compact letter displays of groups.

## 3 RESULTS

### 3.1 *BdIRI* gene analysis

The *Brachypodium* reference genome is predicted to encode six full‐length *IRI* genes (*BdIRI1*, *BdIRI3*, *BdIRI4*, *BdIRI5*, *BdIRI6*, and *BdIRI7*) and one gene (*BdIRI2*) with a truncated protein (based on the v3 RefSeq annotation, GCF_000005505.3); however, this is likely a frameshift mutation as other accessions in Phytozome (Goodstein et al., 2012) do not show this, and thus, it is assumed that normally *Brachypodium* has seven full‐length *IRI* genes. Alignments of all amino acid sequences corresponding to *BdIRI1‐7* using Clustal Omega showed annotated apoplast localization signal sequences, LRR motifs of LxxL where x represents a non‐conserved residue, putative protease hydrolysis sites, and AFPs with ice‐binding motifs of NxVxG/NxVxxG where x represents an outward‐facing residue of the beta‐barrel structure (Figure S1). The genes are found in three gene clusters on chromosome five (Figure S2). The organization shows an adjacent position of two to three genes, with transcription in the same direction within each cluster, and flanked by distinct genes including some that may be involved in epigenetic regulation. It is possible that adjacent *BdIRI* loci are similarly spatio‐temporally or developmentally regulated. For example, *BdIRI3* and *BdIRI4* are adjacent in the genome, and peptides corresponding to the AFP domain of both of these genes were identified after mass spectrometry of CA *Brachypodium* leaves (Bredow et al., 2016).

**FIGURE 1:**
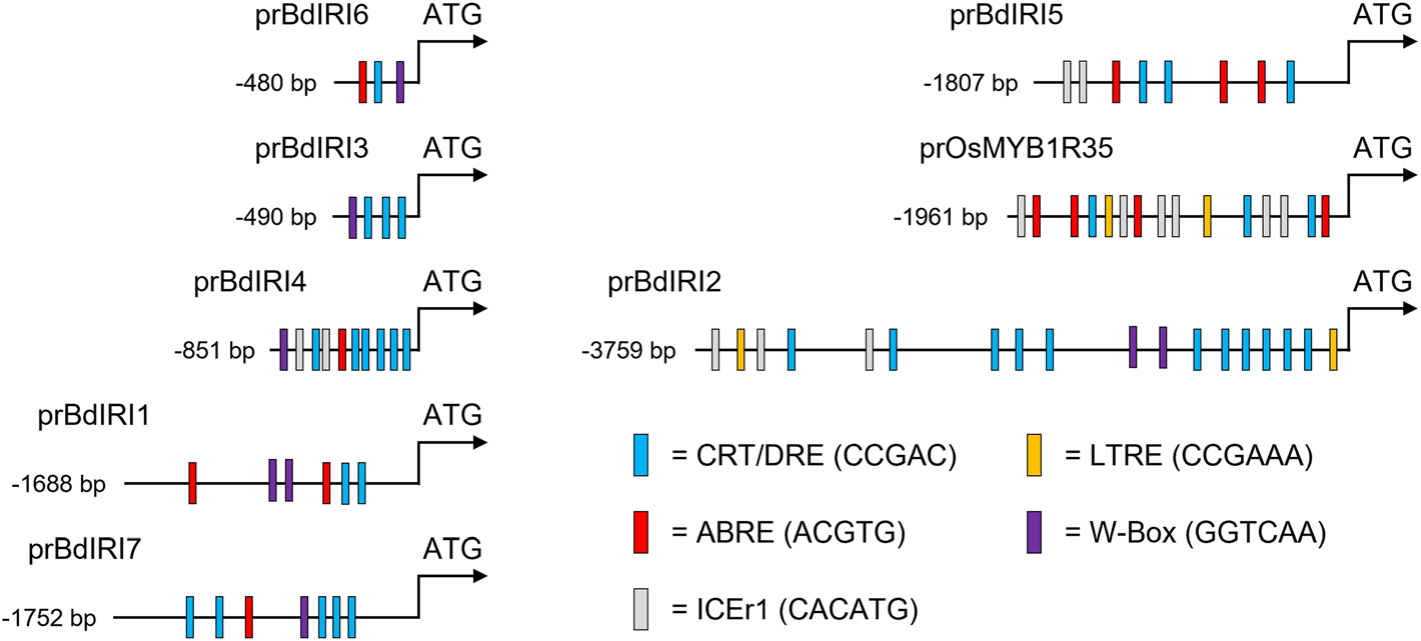
Illustrations of putative promoter regions 5′ of the ATG start codon in the *Brachypodium distachyon* antifreeze protein (AFP) genes *BdIRI1‐7* and the known cold‐regulated promoter sequence of the rice, *Oryza sativa* , gene *OsMYB1R35*. The analysis extended until the stop codon of the nearest upstream gene. *Cis*‐regulatory elements are annotated with strand positions shown relative to the sequence encoding the ATG start. Colored boxes correspond to canonical cold response related and drought‐resistant element motifs (CRT/DRE; blue), the stress hormone, abscisic acid‐responsive elements (ABREs; red), the cold response pathway inducer of C‐repeat binding factor expression 1 (ICEr1; gray), low‐temperature response elements (LTREs; yellow), and the WRKY stress transcription factors recognition W‐box motifs (purple).

To obtain insight into the regulation of the *Bd*AFP genes to better devise a knockdown strategy that targeted AFP expression only after exposure to low temperature, sequences upstream of the translational start site of the *BdIRI*s, as well as the promoter region from a known cold‐inducible gene from rice, pr*Os*MYBR1R35 (Figure 1; Li et al., 2017), were examined for putative *cis*‐acting regulatory elements (CAREs) using PlantCARE (Figure 1; Table S1). All the sequences contained canonical promoters or enhancers such as TATA‐boxes and CAAT‐boxes, as expected. More notably, cold response‐related and drought‐resistant (CRT/DRE) core motifs (CCGAC), which are *cis*‐elements involved in low‐temperature stress responses, were associated with all the promoters. Additional motifs associated with stress and low‐temperatures including various abscisic acid‐responsive elements (ABREs), low‐temperature response elements (LTREs), inducer of cold or C‐repeat binding factor expression 1 CBF expression 1 (ICEr1), drought response elements (DREs), and the WRKY stress transcription factors recognition W‐box motifs were identified and annotated (Figure 1). The cold‐inducible rice promoter shared with the *Bd*AFP promoters multiple CRT/DRE motifs in addition to other elements involved in low temperature regulation and thus the heterologous promoter was deemed suitable to drive expression of the inhibitory miRNA.

The psRNATarget miRNA tool was used to predict endogenous miRNA binding to putative *BdIRI* promoter sequences and the 1961‐bp pr*Os*MYBR1R35 as well as *BdIRI* mRNAs. The algorithm indicated that all of the *BdIRI*s had at least one predicted miRNA target in the corresponding transcript, and all but *BdIRI4* showed miRNA binding sites upstream of the coding region (Table S2). Notably, the 1961‐bp rice promoter region also showed multiple *Brachypodium* miRNA target sites, as did sequences corresponding to *BdIRI1*, *−2*, *−5*, and *−7* gene promoters (Table S2). The observation that the low temperature‐regulated promoter regions for the genes from both species shared some *Brachypodium* miRNA target sites (e.g., miR1583 and miR5174d), again suggested that the choice of this rice promoter to drive expression of the artificial miRNA in *BdIRI* knockdown lines was likely appropriate.

### 3.2 Promoter function and developmental phenotypes

In an attempt to curtail the pleiotropic effects that were likely due to the constitutive expression of the artificial miRNA in previous knockdown constructs (Bredow et al., 2016), CaMV 35S was substituted with the cold‐induced rice promoter. After construction of the plasmids, *Brachypodium* was successfully transformed using a seed cut method (modified from Fursova et al., 2012), which from a total of 150 seeds yielded three and two PCR‐positive transformants for the miRNA and eGFP constructs, respectively. The rapid genotyping method (Ben‐Amar et al., 2017) proved effective and overall, 65% of the recovered T_1_ generation were resistant to the hygromycin‐selective media.

Once homozygous lines were selected, the ability of the heterologous rice promoter to direct transcription was assessed using western blots with transgenic control *Brachypodium* bearing the pr*OsMYB1R35*:eGFP construct (Figure S3). No bands corresponding to GFP were detected in NA or CA wild type extracts, nor in NA pr*OsMYB1R35*:eGFP leaves. However, CA pr*OsMYB1R35*:eGFP extracts showed a band at 26 kDa that co‐migrated with purified GFP. This demonstrates the successful expression of a marker protein in *Brachypodium* driven by the *OsMYB1R35* promoter from *O. sativa*.

**FIGURE 2:**
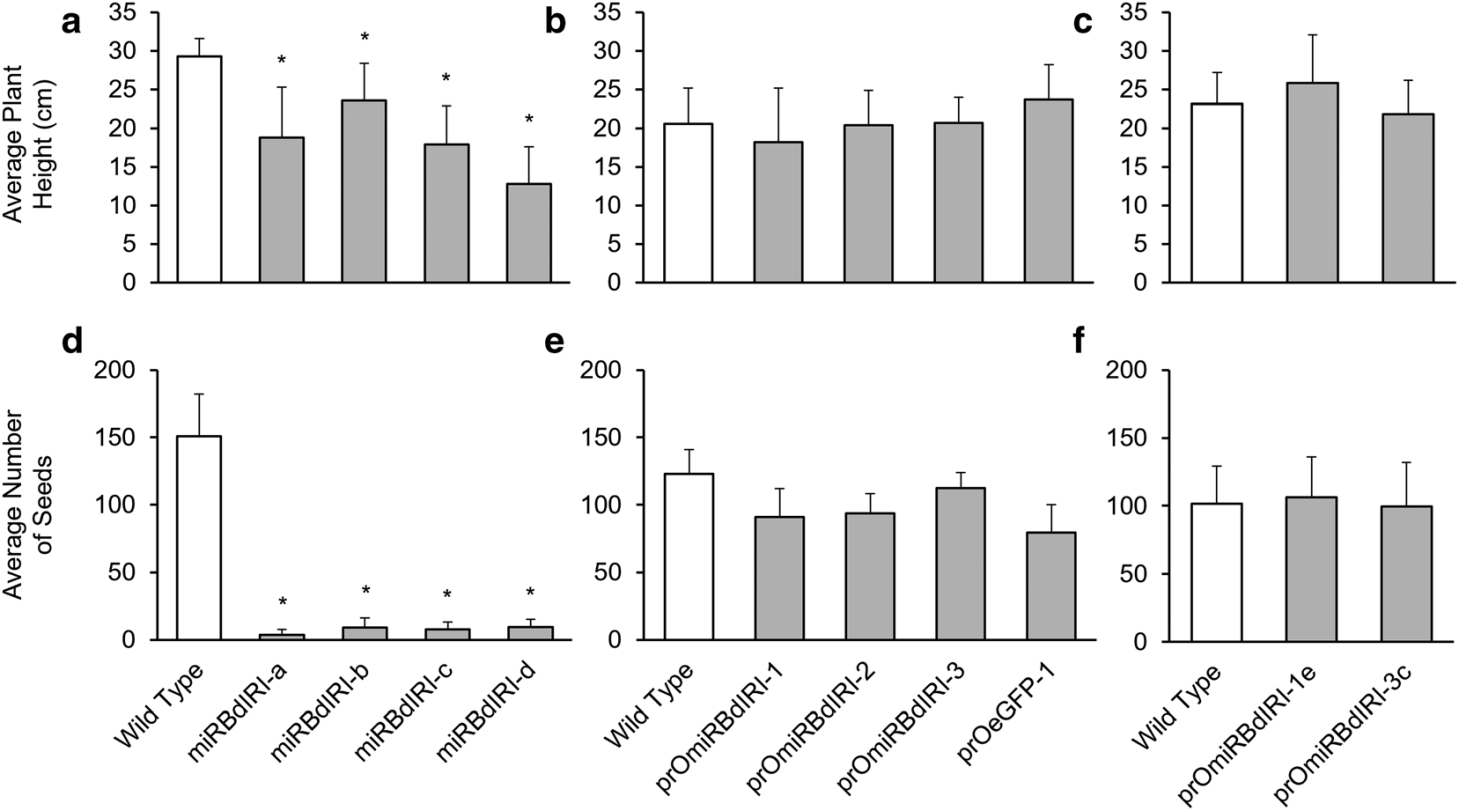
The average height and number of seeds per plant in *Brachypodium* Bd21 wild type plants and plants from transgenic lines. (a,d) Homozygous transgenic lines employing the CaMV 35S promoter ligated to the miRNA sequence to generate constitutively expressed *BdIRI* knockdown plants (data taken from Bredow et al., 2016). (b,e) Heterozygous temporal knockdown lines employing the *OsMYB1R35* promoter and (c,f) homozygous temporal knockdown lines. The data in b, c, e, and f was compiled at 12 weeks from three independent growth trials using at least 15 plants per trial for each knockdown line and wild type. Asterisks indicate significant differences compared to wild type (unpaired *t* test, *p* < 5 × 10^−9^).

Plants bearing the rice promoter ligated to the artificial miRNA sequence appeared to develop similarly to wild type and showed normal phenotypes with respect to height and seed production (Figure S4). Two homozygous lines were identified and designated prOmiRBdIRI‐1e and prOmiRBdIRI‐3c. The prOmiRBdIRI‐1e and prOmiRBdIRI‐3c lines were 25.8 ± 4.1 and 21.9 ± 4.4 cm and set 106.2 ± 29.9 and 99.5 ± 32.6 seeds, respectively, which was not significantly different from wild type at 23.1 ± 4.1 cm and 101.4 ± 27.7 seeds (unpaired *t* tests; assessed at 12 weeks using three independent growth trials with at least 15 plants per trial; Figure 2). Germination rate was also the same at 90.4 ± 10.9%, 90.0 ± 13.1%, and 91.25 ± 11.3%, for wild type, prOmiRBdIRI‐1e, and prOmiRBdIRI‐3c lines, respectively (8 independent growth trials using at least 10 seeds per trial).

### 3.3 Antifreeze activity and freeze resistance

**FIGURE 3:**
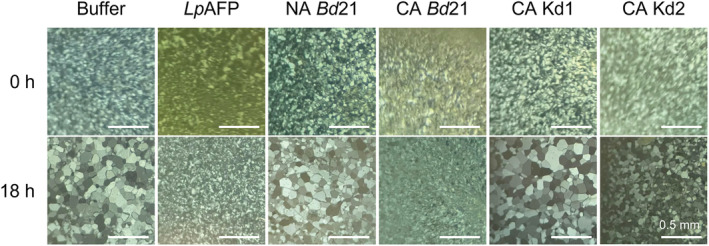
Representative ice recrystallization inhibition “splat” assays of apoplast extracts using non‐acclimated (NA) and cold‐acclimated (CA) *Brachypodium distachyon* Bd21 wild type and temporal antifreeze protein (AFP) knockdown lines prOmiRBdIRI‐1e (Kd1) and prOmiRBdIRI‐3c (Kd2). Samples were annealed at −6°C for 18 h at a standardized concentration of 0.01 mg ml^−1^. Buffer and recombinant purified rye grass *Lolium perenne* AFP (*Lp*AFP) controls are also shown. Assays were performed in triplicate with similar results, and representative images are shown. Scale bars represent 0.5 mm. Splat assays performed using CA prOmiReGFP plants resulted in results similar to those obtained using wild type Bd21, indicating that the presence of the plasmid did not affect AFP activity (not shown).

The reduction of AFP activity in the knockdown lines mediated by the translation inhibitory miRNA (Bredow et al., 2016) was tested using a variety of assays. “Splat” assays were used to visualize IRI activity in crude extracts or apoplast samples (Figure 3, Figure S5). CA knockdown prOmiBdIRI‐1e and prOmiRBdIRI‐3c lines showed reduced IRI activity compared to wild type CA plants and were similar to assays of NA wild type and NA transgenic lines. A dilution series used to estimate the difference in activity showed that at 0.01 mg mL^−1^, CA knockdown lines and NA wild type showed larger ice crystals at the conclusion of the annealing period compared to samples from CA wild type and CA prOmiReGFP (Figures 3 and S5). Thus, CA appears to regulate the rice promoter to drive the miRNA to attenuate the expression of the AFP gene products. It is possible that endogenous *BdIRI* promoters may have some very low levels of expression under NA conditions but the comparison with CA conditions was clear. As well, although the knockdown lines both show attenuation of AFP activity, there appeared to be minor differences in expression, undoubtedly due to position effects, as has been shown in other transgene insertions (e.g., Duan et al., 2008; Liu et al., 2017; Simón‐Mateo & García, 2006).

AFP levels as assayed using TH were virtually undetectable in NA samples but were significantly higher at 0.05°C in wild type tissue extracts from CA plants (Table 1; Figure S6). Plant AFPs have TH levels that are typically low with even purified *Bd*AFPs previously reported at only 0.08°C (Bredow et al., 2016). CA transgenic prOmiRBdIRI‐1e and prOmiRBdIRI‐3c had TH activities that were significantly lower (77% and 74%) than the levels in CA wild type plants. Strikingly, these low temperature induced transgenic knockdowns showed levels of TH at 0.013°C and 0.012°C, comparable with previously reported constitutive knockdowns expressing the same miRNA, at a TH range of 0.009–0.034°C (Table 1 and Bredow et al., 2016), confirming the successful silencing of *Bd*AFP activity even when the artificial miRNA was driven by the temporal rice promoter.

**TABLE 1:** Thermal hysteresis (TH) readings for crude protein extracts from leaf tissue lysates on cold‐acclimated (CA) and non‐acclimated (NA) Bd21 wild type and temporal cold‐induced antifreeze protein knockdown lines prOmiRBdIRI‐1e and prOmiRBdIRI‐3c. Samples were tested at 40 mg ml^−1^ of total protein concentrated from crude cell extracts. Readings were captured using a nanoliter osmometer. Assays were performed in triplicate and values shown are the average of three replicates shown with standard deviation.

Sample	TH (°C)
Buffer	0
Wild type, Bd21 (CA)	0.050 ± 0.019
Wild type, Bd21 (NA)	0.005 ± 0.003
prOmiRBdIRI‐1e (CA)	0.013 ± 0.002
prOmiRBdIRI‐1e (NA)	0.003 ± 0.003
prOmiRBdIRI‐3c (CA)	0.012 ± 0.011
prOmiRBdIRI‐3c (NA)	0.002 ± 0.002

*Brachypodium* AFPs shape ice into hexagon crystals followed by a smooth, irregular, flower‐shaped burst (Figure S7). As would be expected, samples from CA wild type plants showed obvious ice shaping, but some minimal shaping still occurred in NA wild type and knockdown samples (Figure S5), consistent with the IRI and TH activity results. Ice shaping in the CA knockdowns appeared to occur on the primary prism plane, favoring slight shaping along the *a*‐axis before weakly bursting (Figure S5), again consistent with low activity. In comparison, the CA wild type showed initial adsorption affinity for the *a*‐axis primary prism plane quickly followed by adsorption and shaping on the *c*‐axis basal plane, forming obvious stunted hexagonal bipyramidal forms with a strong burst when the freezing point was exceeded.

**FIGURE 4:**
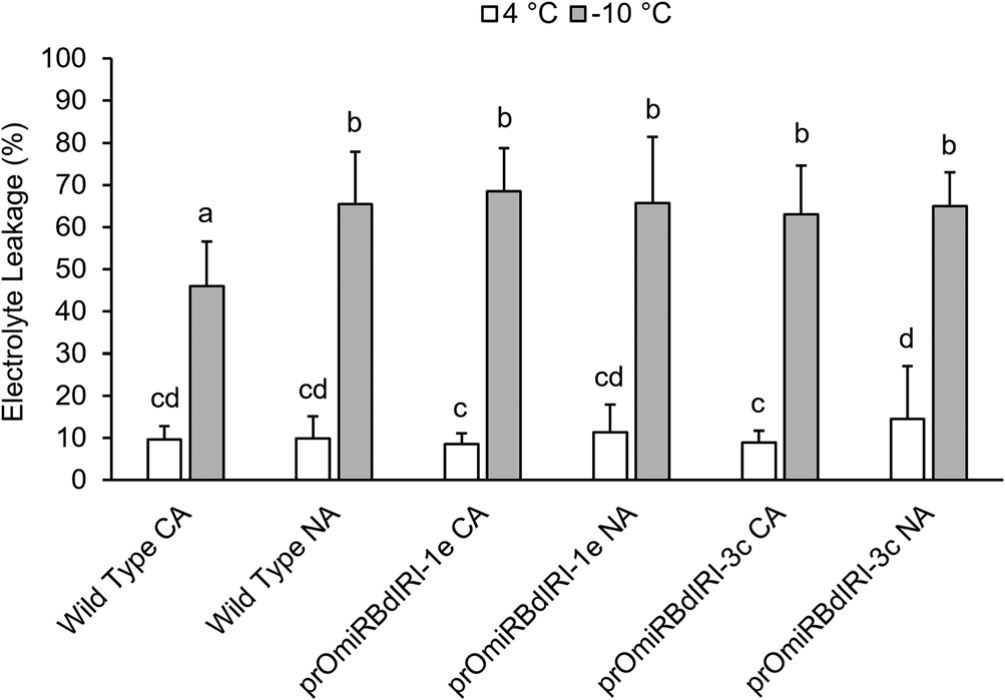
Electrolyte leakage assays performed on non‐acclimated (NA) and cold‐acclimated (CA) wild type *Brachypodium distachyon* Bd21 and two homozygous temporal antifreeze protein knockdown lines (prOmiRBdIRI‐1e and prOmiRBdIRI‐3c). Control leaves were maintained at 4°C (white bars), whereas experimental samples were incubated at −10°C (gray bars) as indicated. Electrolyte leakage was measured as a percentage of electrolytes released after the freeze protocol as a ratio of the total released electrolytes after autoclaving, based on the total leaf mass in the sample (see Section 2). Letters represent statistically significant groups following one‐way analysis of variance (ANOVA) with post‐hoc Tukey multiple test correction (*p* < .05). Error bars represent the standard deviation of the mean, and assays were performed in triplicate (*n* = 10).

Rounded *Bd*AFP‐mediated ice burst morphology presumably would help protect membranes by preventing the growth of large, sharp ice crystals as is seen in some freeze‐avoiding organisms (Bar Dolev et al., 2016). Such membrane protection can be quantitatively assessed by electrolyte leakage assays. The CA transgenic leaves showed a significant increase (~30%) in electrolyte leakage (*p* < .05, one‐way analysis of variance [ANOVA]) at −10°C when compared with CA wild type (Figure 4). At higher freezing temperatures of −6°C, electrolyte leakage was variably increased in the CA knockdown lines relative to CA wild type leaves (not shown). In all experiments, leaves from plants maintained at 4°C showed relatively little electrolyte leakage independent of the genotype, as expected.

**FIGURE 5:**
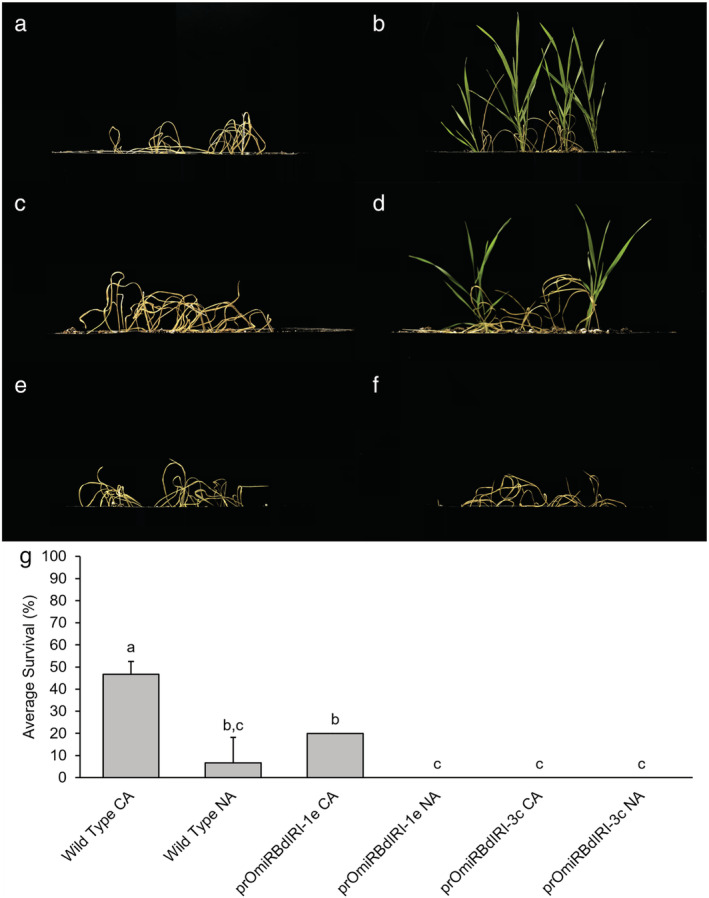
Representative whole plant freezing survival assay of non‐acclimated (NA) and cold‐acclimated (CA) wild type *Brachypodium distachyon* Bd21 and two homozygous knockdown lines (prOmiRBdIRI‐1e and prOmiRBdIRI‐3c). (a) NA Bd21 wild type. (b) CA Bd21 wild type. (c) NA prOmiRBdIRI‐1e. (d) CA prOmiRBdIRI‐1e. (e) NA prOmiRBdIRI‐3c. (f) CA prOmiRBdIRI‐3c. (g) Values represent survival and are the average of three replicates, with error bars showing the standard deviation of the mean. Letters represent statistically significant groups following one‐way analysis of variance (ANOVA) with post‐hoc Tukey multiple test correction (*p* < .01). Two‐week‐old plants were frozen at 1°C h^−1^ until a final temperature of −8°C in a controlled chamber in the dark following misting with sterile water to initiate freezing. Plants were allowed to recover for 2 days at 4°C with no light and returned to standard conditions for 7 days before images (a–f) were captured. Assays were performed in triplicate (*n* = 10).

**FIGURE 6:**
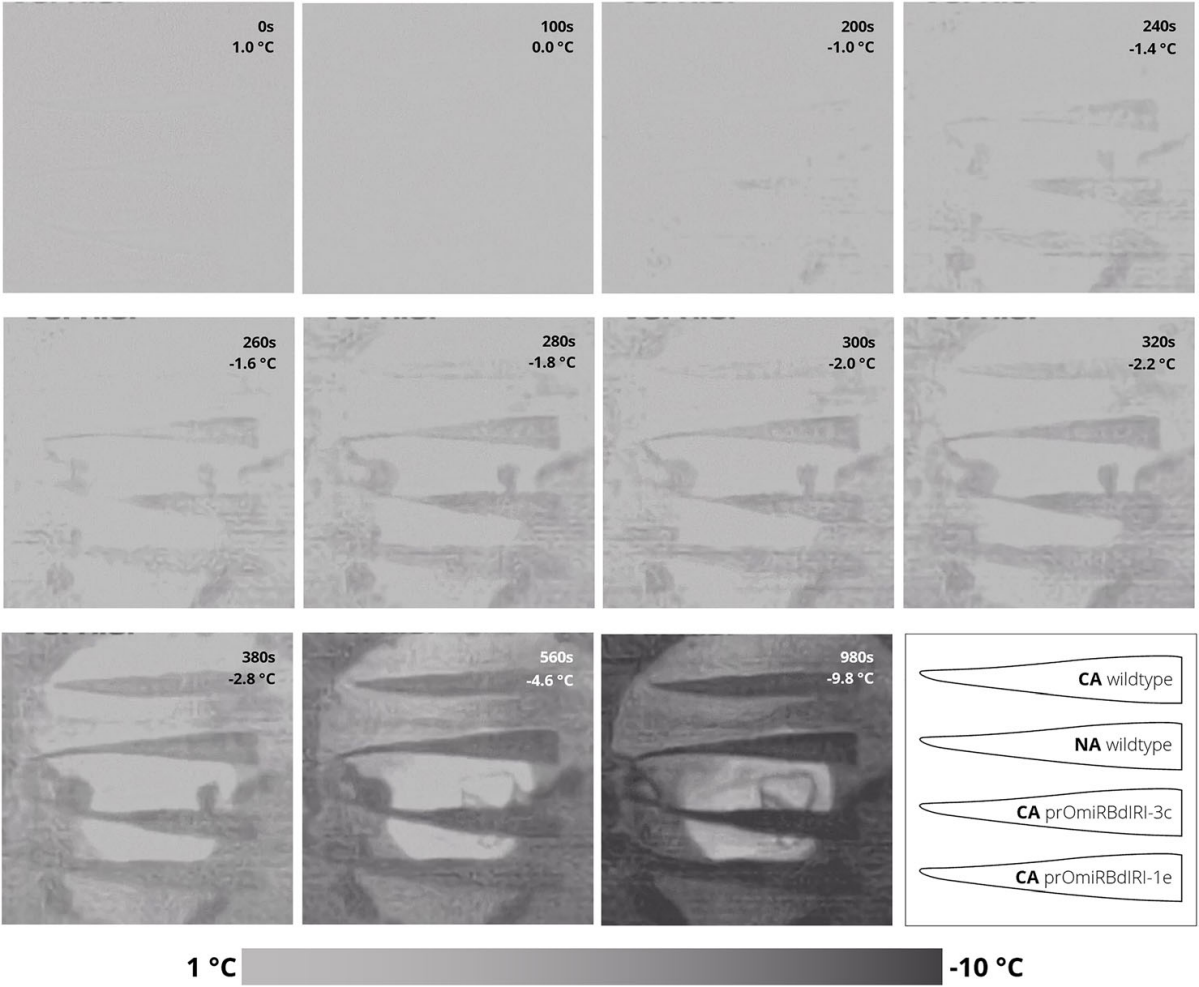
Representative thermographs of excised leaf tissue of wild type cold‐acclimated (CA), wild type non‐acclimated (NA), prOmiRBdIRI‐1e CA, and prOmiRBdIRI‐3c CA, from top to bottom, respectively. Leaves were equilibrated at 1°C and frozen to −10°C. Ice propagation was nucleated by an ice chip placed in 10 μl of water on the excision wound. Time stamps and temperatures are indicated. A diagram of the leaf samples is shown in bottom right with a temperature scale shown at the bottom.

When entire plants were frozen to −8°C, 47% of CA wild type survived, significantly more (*p* < .01, unpaired *t* test) compared with 20% of CA prOmiRBdIRI‐1e plants and none of the CA prOmiRBdIRI‐3c plants (Figure 5). The differences in the knockdown lines may again reflect minor differences in miRNA‐mediated translational attenuation of *BdIRI* mRNAs due to position effects, as noted earlier. Importantly, none of the NA plants survived, independent of genotype, and suggested that even any “leaky” AFP activity was insufficient to impact survival at −8°C. To further assess ice propagation and freezing patterns of the temporal knockdown compared to wild type plants, infrared thermography was used as encouraged by previous observations of ice nucleation and propagation in various species (Ball et al., 2002; Lutze et al., 1998; Sekozawa et al., 2004; Wisniewski et al., 1997; Wisniewski et al., 2015). Images of CA and NA freezing leaf tissue from wild type compared with knockdown lines as the temperature was reduced to −10°C were distinct, suggesting that AFPs can slow the propagation of ice through leaf tissues and were consistent with the electrolyte leakage assays (Figure 6). Thermographs further supported the conclusion that ice propagation at subzero temperatures was more rapid in leaves from AFP knockdown lines. Temperature readings collected concurrently with thermographs on leaves show CA wild type leaves were 1–2°C warmer than the NA wild type and CA knockdowns, in agreement with the known ~2°C freezing point depression of INPs mediated by *Bd*AFPs with a divergence around −2°C where INPs nucleate ice (Figure S8).

**FIGURE 7:**
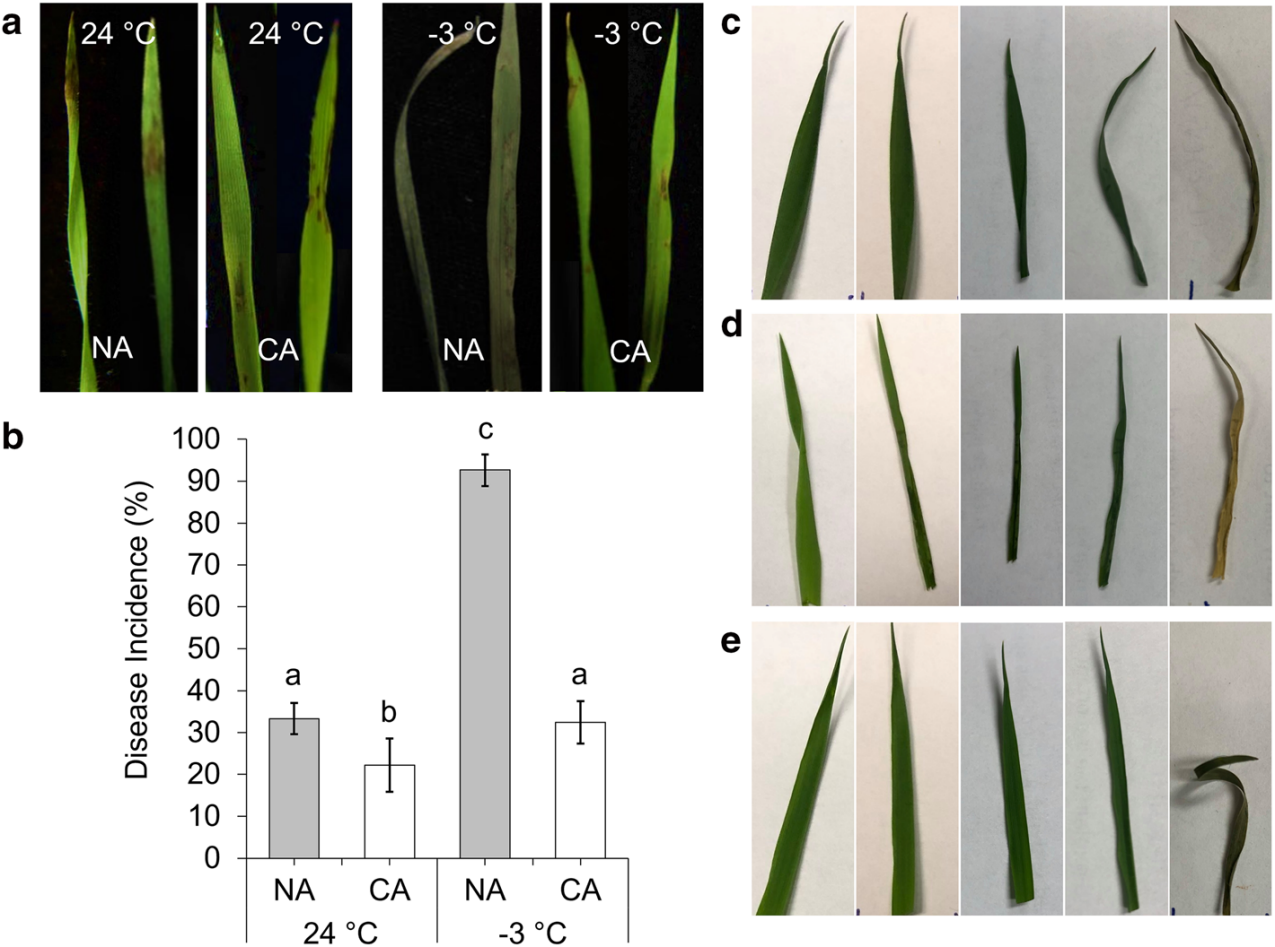
Ice nucleation activity and infection of *Brachypodium distachyon* with *Pseudomonas syringae* pv. *syringae* B728A. (a) Portions of leaves from non‐acclimated (NA) and cold‐acclimated (CA) plants sprayed with C.A. *P*. *syringae* pathovar cultures at 24°C standard conditions (the pair of left images) and −3°C (the pair of right images) showing disease incidence, and in the case of the NA leaves at −3°C, freeze damage. (b) Disease incidence measured as a percentage of leaves sprayed with the C.A. *P. syringae* pathovar showing disease symptoms with leaves from plants that were either NA and CA and then infected as whole plants and kept at the two temperatures shown. (c) Excised *Brachypodium distachyon* leaves infected with cold activated *Pseudomonas syringae* pv. *syringae* B728A, with the five images (left to right) representing pre‐infection, following infection and incubation for 12 h at −3°C, and during recovery at 4°C at 24 h post infection, 48 h post infection, and 1 week post infection showing infected cold‐acclimated (CA) Bd21 wild type, (d) infected CA induced antifreeze protein (AFP) knockdown line prOmiRBdIRI‐1e, and (e) uninfected CA Bd21 wild type controls. As indicted in the Section 2, the bacterial strain was cultured at 28°C to OD_600_ = 0.6–1.0 and placed at 4°C for 2 days before resuspending in 10‐mM MgCl_2_ and diluted to OD_600_ = 0.2 to an approximate concentration of 1 × 10^8^ colony forming units ml^−1^ prior to infection by dipping wounded ends of leaves in cultures. Assay was performed in triplicate with similar results and also performed with leaves from non‐acclimated plants (not shown).

Ice nucleation under natural conditions is invariably initiated by INA+ bacteria, including strains of *P. syringae*, but as noted, *Brachypodium* AFPs can attenuate INPs in vitro (Bredow et al., 2018). To measure the impact of *Bd*AFPs on pathogen infection, wild type and a knockdown line were exposed to *P. syringae* pv. *syringae* B728A, a pathovar with INPs (Feil et al., 2005). Leaves from NA wild type and CA knockdown lines treated with the bacterial culture and placed at −3°C, a temperature below which this pathovar is known to nucleate ice (Figure S9A), displayed disease‐like symptoms including water soaking and cell death, whereas infected plants that were not subjected to freezing temperatures displayed non‐freeze‐associated infection symptoms 12 h post infection (Figures 7a,b and S9B‐S9C). In contrast, CA wild type leaves with AFP activity displayed little evidence of tissue damage (Figure 7d–f). Furthermore, after 24–48 h post infection, leaves from plants known to have little or no AFP activity were shriveled and dry compared with CA wild type leaves, likely due to cell lysis associated with ice nucleation and subsequent ice growth. These same leaves showed more disease symptoms and cell death 1 week after pathovar exposure compared with CA wild type controls. Although it is not surprising that treated CA wild type leaves showed symptoms of disease considering the bacterial titre used, they appeared to have greater resistance to dehydration compared with the leaves with low AFP activity. This qualitative assay suggests that the presence of sufficient AFPs in the CA wild type plants can help protect against ice‐mediated membrane damage and ameliorate the impact of pathogens with INA activity.

## 4 DISCUSSION

### 4.1 Shared putative MicroRNA targets in the Rice and *Brachypodium* sequences

Plant gene expression can be regulated by miRNAs both transcriptionally (Yang et al., 2019) and post‐transcriptionally (Bertolini et al., 2013; Jones‐Rhoades et al., 2006; Mallory & Vaucheret, 2006; Zhang, 2015), with the cold‐stress response of *Brachypodium* being no exception (Zhang et al., 2009). Among monocots, *Brachypodium* and rice have the highest number of annotated miRNAs at 525 and 713 (*O. sativa*) in the miRBase database (Release 21). Again, to inform the knockdown strategy, all 7 *BdIRI* genes, as well as the rice promoter, pr*Os*MYBR1R35, were analyzed for potential miRNA binding sites. Within the *BdIRI* coding regions, the majority of miRNA sites (14/28) belong to the bdi‐miR395 family that has homologs in rice as well as *Arabidopsis* (Zhang et al., 2009). This miRNA family is stress‐regulated with members known to target disease‐resistant proteins (Baev et al., 2011; Fujii et al., 2005; Jones‐Rhoades & Bartel, 2004; Lv et al., 2016). Other miRNAs from the miR169 family had 6 putative targets in *BdIRI2* and *BdIRI5* and are likely involved responses to oxidative stress (Lv et al., 2016). Likewise, miRNAs are predicted to regulate multiple *BdIRI*s including bdi‐miR7717a‐5p on *BdIRI2* and *BdIRI3* and bdi‐miR5055 on *BdIRI2*, *BdIRI3*, *BdIRI5*, and *BdIRI6*, suggesting that *BdIRI*s can be regulated by common miRNAs, in addition to others that target individual *BdIRI* transcripts. For example, *BdIRI4*, *−3* and *−1* are clustered on the chromosome, but only *Bd*AFP isoforms 4 and 3 were found after leaf mass spectrophotometric analysis (Bredow et al., 2016), suggesting that the absence of *Bd*AFP isoform 1 may be due to miRNA regulation by bdi‐miR159b‐3p.2 which has a binding site in the *BdIRI1* but not in the other *BdIRIs*.

### 4.2 Heterologous promoter‐driven expression

The selection of an appropriate promoter to drive transcription in transgenic organisms is frequently challenging. Where libraries of promoters and enhancers are available, such as the rich resources available for *Drosophila* geneticists, transcription of a coding sequence of interest can be finely tuned. For grasses, genetic resources are scarcer, and in the absence of annotated promoter sequences, use of constitutive promoters such as CaMV 35S can risk inappropriate expression, epigenetic silencing, and even suboptimal growth (Amack & Antunes, 2020; Estrada‐Melo et al., 2015; Rajeevkumar et al., 2015). For example, constitutive expression of an artificial miRNA that targets the translation of *BdIRI* gene products resulted in short stature, low germination rate, and near‐sterility in transgenic plants (Bredow et al., 2016). As an alternative to constitutive promoters, synthetic promoters consisting of core promoters and combinations of enhancer sequences for regulatory elements, sometimes from heterologous species, can be constructed with uncertain outcomes (Ali & Kim, 2019; Mohan et al., 2017). Again, as another somewhat risky strategy, entire promoter sequences from evolutionarily related plants may allow for a greater degree of control including spatio‐temporal expression (Dutt et al., 2014) and can even be free from endogenous signaling that could lead to undesired effects with native host promoters (Napoli et al., 1990). As noted, non‐coding RNAs including miRNAs can influence expression with roles in developmental regulation and stress responses (Budak & Akpinar, 2015; Chen, 2009; Liu et al., 2017; Peter, 2010; Waititu et al., 2020; Wu et al., 2010). Complementary sequence targets include gene transcripts that can be hydrolysed, translationally inhibited, or can localize to the nucleus and target promoter sequences (Jones‐Rhoades et al., 2006; Li et al., 2013; Yang et al., 2019). Their frequent roles in the regulation of stress responses are of interest with regard to CA genes. The *Brachypodium* Poaceae lineage diverged from rice ~50 million years ago, with high homology and synteny between *Brachypodium* and rice genomes (Bossolini et al., 2007; Huo et al., 2009; International Brachypodium Initiative [IBI], 2010; Kumar et al., 2009). Thus, the identification of predicted *Brachypodium* miRNA target sequences in both *BdIRI* as well as the *OsMYB1R35* rice promoter that is induced at 4°C (Li et al., 2017) suggests an evolutionarily conserved low temperature response. This, in turn, encouraged the prospect that the rice promoter would not only be recognized in *Brachypodium* but could be appropriately regulated by any of the identified *Brachypodium* miRNAs with target sites in the rice sequence (Tables S1 and S2). Here, we show that the use of this rice promoter indeed functioned in *Brachypodium*, and there were no obvious detrimental phenotypes associated with its use, either in the transgenics bearing the artificial miRNA sequence or in eGFP expression plants (Figure 2).

### 4.3 AFP knockdowns and their vulnerability to freezing and *P. syringae*

As noted, the generated transgenic lines appeared to have no obvious developmental defects and as such the observation that they displayed similar freeze susceptibility as those lines bearing a constitutive promoter to drive the miRNA‐mediated attenuation of AFPs convincingly demonstrated that these proteins contribute to freeze tolerance. Thus, these observations are consistent with experiments that showed that the addition of AFPs to susceptible plants provided some low temperature protection (e.g., Wallis et al., 1997; Khanna & Daggard, 2006). In wild type *Brachypodium*, a short 2‐day CA period is both necessary and sufficient to prepare the plants for survival to subzero temperatures and is coincident with the appearance of AFPs. Although *Bd*AFPs show low TH, depressing the freezing point of solutions only marginally, more importantly, the results support the AFP‐mediated attenuation of pathogen‐mediated INA, the shaping of ice, the restriction of ice crystal growth as measured by IRI, the reduction of electrolyte leakage, and ultimately facilitate whole plant survival even after freezing. Especially striking was the killing of all plants in one line and a second line showing less than half the survival rate compared with wild type controls at −8°C. All these AFP‐related properties were effectively knocked down by the temperature‐regulated response of the rice promoter to direct miRNA expression (Figures 3, 4, 5).

It has been assumed that AFPs are associated with the plasma membrane as reports of a fish AFP bound to model lipid bilayers changed phase transition and prompted researchers to recommend the use of fish AFPs to confer low temperature survival to plants, which ultimately was not met with success (Kenward et al., 1993; Kenward et al., 1999; Tomczak et al., 2002). In plants, the localization of AFPs in the apoplast would suggest limited access to plasma membranes in any case. Indeed, mass spectrophotometry of CA *Brachypodium* plasma membrane proteins did not reveal any *Bd*AFPs (Juurakko, Bredow, et al., 2021). Nonetheless, AFPs do protect plasma membranes from damage, presumably from uncontrolled ice growth initiated by nucleators in the apoplast with its low solute concentration, and across cell walls into the cytoplasm.

The apoplast location of the AFPs allowed their assay in the absence of many other contaminating proteins and yielded clear evidence of the knockdown of IRI activity in the CA transgenic lines with the appearance of large ice crystals after the annealing period (Figure 3). Activity in lysates was consistent but not as visually clear, although individual ice crystals were substantially larger in dilute samples from knockdowns compared with controls. As judged by western blot analysis of samples from transgenic plants expressing eGFP, the cold‐induced rice promoter was not strong in *Brachypodium*. Thus, it is somewhat surprising that after a 2‐day induction period, the heterologous promoter‐directed miRNA lines showed AFP activities similar to the earlier‐reported constitutively expressed miRNA lines with respect to IRI, electrolyte leakage, whole plant freezing survival, and TH (Figures 3, 4, 5, and S6 vs. Bredow et al., 2016). This suggests that the single artificial miRNA designed to complement the multiple *BdIRI* transcripts was very effective notwithstanding the heterologous promoter and the miRNA's less than perfect “match” and underscoring the effectiveness of knockdown by translational attenuation (Bredow et al., 2016).

Structural ice barriers and thermal decoupling controlled by plant architecture and morphology, well‐known in angiosperms between stems and flowers, may not be as easily applied to grasses (Bertel et al., 2021; Kuprian et al., 2014). No evidence of either was observed in freezing *Brachypodium* leaves analyzed by infrared thermography. However, the presence of AFPs in CA wild type tissue was coincident with the obvious retarded advancement of freeze fronts, a lag that was on average 1–2°C different in leaves with AFPs than without, and similar to the ~2°C attenuation of ice nucleation activity by *Bd*AFPs (Bredow et al., 2018, Figure 7). There was no inhibition of ice front development observed in NA wild type and CA AFP knockdown lines suggesting that AFPs can delay ice propagation in leaf tissue, consistent with IRI, electrolyte leakage, and whole plant freezing assays. As mass spectrophotometric analysis has shown that *Bd*AFPs encoded by *BdIRI3* and *4* were detected in leaf tissue (Bredow et al., 2016), at least these two isoforms are associated with leaf ice growth protection. Adsorption of the AFPs to forming ice crystals will keep crystals small and less damaging to adjacent membranes and thus limiting leaf damage.

Similar to the reduction of freezing in leaves by AFPs was the retardation of damage following infection as seen after exposure of wounded leaves to *P. syringae* pv. *syringae* B728A. This pathovar can nucleate ice at high subzero temperatures presumably as a means to destroy tissues and access nutrients (Feil et al., 2005). As noted, *Bd*AFPs inhibit the activity of *P. syringae*'s INP in vitro (Bredow et al., 2018), and we speculate that a physical interaction of the INP and *Bd*AFPs is sufficient to impact bacterial fitness, such that the progress of infection was slowed down. This effect was seen as early as 12–48 h after the pathogen challenge and with the knockdowns showing more cellular death 1‐week post infection. Thus, it would be of interest in the future to more fully explore the relationship between AFP activity and the infectivity of pathogens bearing INPs.

The cause of the developmental effects seen after the use of the constitutive promoter to drive miRNA expression (Bredow et al., 2016) is unknown. One potential reason may be the simultaneous knockdown of the adjoining LRR domains from the *BdIRI* primary translation products. In general, LRRs function in protein–protein interactions and have roles in growth and development including LRR‐receptor kinases that regulate plant growth and development and LRR‐extension proteins, which function in growth as well as pollen tube formation (Fischer et al., 2016; He et al., 2018; Zhao et al., 2018). However, to our knowledge, the characterization and function of plant LRR domains associated with AFPs have not been reported, and any impact of their knockdown during development is unexplored. Instead, the defective phenotypes may have simply arisen due to the constitutive‐driven artificial miRNA expression non‐specifically interfering with gene regulation and polysome loading during critical developmental stages, or alternatively, the possibility that AFPs have an as yet undiscovered role in *Brachypodium* development. Whatever the explanation, however, the difficulty was effectively ameliorated by employment of the CA rice promoter.

### 4.4 Conclusions and future prospects

Taken together, we have shown for the first time that the substitution of a heterologous temporally regulated promoter to direct the expression of an artificial miRNA that targets the *BdIRI* transcripts resulted in no apparent developmental defects but nevertheless substantially reduced AFP activity. Not only were these lines more susceptible to low temperatures, but they also showed increased vulnerability to a low‐temperature associated phytopathogen. In consequence, these results highlight the potential of *Brachypodium* AFPs as candidates for the development of freeze‐tolerant and more pathogen‐hardy horticultural crops, if not food crops where there may be public resistance to genetically modified plants. Additional biotechnological and research applications extend outside agriculture and range from additives to prevent ice recrystallization in stored cells and tissues, processed foods, and pharmaceuticals, particularly where infrastructure for flash freezing and extremely low temperature storage is not viable or otherwise unavailable. This research also opens the way to explore *in planta* the effects of AFPs on freezing tolerance and pathogen susceptibility as well as the means by which *BdIRI*s may be regulated through *cis*‐regulatory elements and endogenous miRNAs.

## CONFLICT OF INTEREST

The authors declare that the research was conducted in the absence of any commercial or financial relationships that could be construed as a potential conflict of interest.

## AUTHOR CONTRIBUTIONS

CLJ conducted all experiments, analyzed all data, and produced all figures. CLJ wrote the initial draft of the manuscript, and all authors contributed to manuscript revision. MB assisted in the design of some early experiments and conducted preliminary infection experiments. VKW and GCD co‐supervised and secured funding.

## Supporting information


**Figure S1.** Alignments performed using Clustal Omega (https://www.ebi.ac.uk/Tools/msa/clustalo/) of all 7 *BdIRI* amino acid sequences from the newest assembly ( *Brachypodium distachyon* genome v3) except that a likely frameshift mutation for *BdIRI2,* which appears in the reference genome but not in other accessions, is not shown here, and neither is the 22 amino acid extension on the N‐terminal in BdIRI4, which is likely a result of an additional or incorrectly noted start codon. The annotated apoplast localization signal sequence is shown in red, LRR motifs of LxxL where x represents a non‐conserved residue in green, and AFP motifs of NxVxG/NxVxxG where x represents an outward‐facing residue of the beta‐barrel structure in blue, along with the putative asparagine endopeptidase hydrolytic cleavage sites indicated by black arrows. Asterisks (*) denote fully conserved residues, colons (:) denote conservative substitutions, and periods (.) denote semi‐conservative substitutions.
**Figure S2.** Illustration showing the three clusters and chromosomal positions of the 7 *BdIRI* genes on chromosome five of *Brachypodium* ( *Brachypodium distachyon* genome v3). *BdIRI* gene numbers are labeled and strands (+/−) are labeled and highlighted. Flanking and other nearby genes are shown with *BdIRI* genes are highlighted in blue. NCBI Entrez Gene IDs are labeled. The dotted line indicates the continuity of the chromosome.
**Figure S3.** Representative western blot analysis for characterization of the rice promoter pr*OsMYB1R35* in *Brachypodium distachyon* using extracts from non‐acclimated (NA) and cold‐acclimated (CA) Bd21 wild type and prOeGFP transgenic plants and visualized with antibodies for green fluorescent protein (GFP). **(A)** Lane 1 corresponds to NA prOeGFP, lanes 2–5 to CA prOeGFP from separate individual plants, lane 6 to purified recombinant GFP used as a positive control, lane 7 to NA wild type, and lane 8 to CA wild type. **(B)** RuBisCO large chain (Rbcl) was used as a loading control with Coomassie Brilliant Blue (CBB) staining. Molecular weights of bands corresponding to eGFP (26 kDa) and Rbcl (55 kDa), are labeled. Western blots were performed in triplicate. Note: positive control recombinant eGFP was overloaded and burnt during imaging.
**Figure S4.** Phenotypes of senescent Bd21 wild type *Brachypodium distachyon* **(A)** plants and two homozygous knockdown lines, prOmiRBdIRI‐1e **(B)** and prOmiRBdIRI‐3c **(C)**, bearing our temporal AFP knockdown systems. Photos were taken at 12 weeks following the described standard growth conditions with water and fertilizer withdrawn in the final week to allow senesced plants to dry out for seed harvesting.
**Figure S5.** Ice recrystallization inhibition “splat” assays of cell protein extracts using leaf tissue from non‐acclimated (NA) and cold‐acclimated (CA) *B. distachyon* Bd21 wild type and temporal AFP knockdown lines prOmiRBdIRI‐1e and prOmiRBdIRI‐3c. Samples were annealed at −6 °C for 18 h at various concentrations. Buffer and *Lp*AFP controls are shown. Scale bar for splat assays represents 0.5 mm. Ice crystal morphologies and burst patterns are shown alongside and were tested at 40 mg mL^−1^ of total protein concentrated from crude cell extracts. Micrographs were captured at 50x zoom on a nanoliter osmometer with scale bars representing 10 μM. All assays were performed in triplicate with similar results and representative images are shown.
**Figure S6.** Thermal hysteresis (TH) readings for crude protein extracts from leaf tissue lysates on non‐acclimated (NA) and cold‐acclimated (CA) Bd21 wild type *Brachypodium distachyon* and two temporal AFP knockdown lines prOmiRBdIRI‐1e and prOmiRBdIRI‐3c. Samples were tested at 40 mg mL^−1^ of total protein concentrated from crude cell extracts. Readings were captured using a nanoliter osmometer and performed in triplicate. Letters represent statistically significant groups following one‐way ANOVA with post‐hoc Tukey multiple test correction (*p* < 0.01).
**Figure S7.** Ice crystal morphology and burst patterns in the presence of antifreeze proteins in crude lysates of wildtype cold‐acclimated *B. distachyon* Bd21 leaf tissue viewed down on the *a*‐axis **(A1‐A3)** and viewed horizontally towards the *a‐*axis **(B1‐B3)**. *Brachypodium* AFPs have an affinity for the prism and basal planes. Buffer solution showing unrestricted ice crystal growth in the absence of AFPs with characteristic disk‐shaped morphology visible, viewed down on the *a*‐axis **(C1‐C3)**. Micrographs were captured at 50x zoom on a nanoliter osmometer and performed in triplicate. Scale bar is 10 μm.
**Figure S8.** Infrared thermography assay performed on cold‐acclimated (CA) wild type (blue), CA prOmiRBdIRI‐1e (gray), CA prOmiRBdIRI‐3c (purple) knockdown lines, and non‐acclimated (NA) wild type (yellow). Data represent the recorded thermography data where each point represents the temperature at a single frame captured at a frame rate of 1 frame every 4 sec. Lines shown represent the moving averages using a period of 25. Points measured were 5 mm from the wounded end of the leaf where the tissue was excised from plants. Leaves were equilibrated at 1 °C for 30 min and frozen to −10 °C at a rate of 0.01 °C sec^−1^. Experiments were performed in triplicate. CA prOmiRBdIRI‐1e, CA prOmiRBdIRI‐3c knockdown lines, and NA wild type were significantly different from CA wild type indicated by stars (one tailed *t*‐test, unpaired).
**Figure S9.** Ice nucleation activity and infection phenotypes for *Brachypodium distachyon* with *Pseudomonas syringae* pv. *syringae* B728A. **(A)** Ice nucleation activity of the *P. syringae* pathovar that had been cold‐activated by transfer to 4 °C for 48 h (C.A.) or kept at 24 °C, representing non‐cold‐activated (N.A.) cultures with nucleation temperatures between −2 °C and −4 °C, were used in assays to determine the conditions and temperature of incubation for leaf tissue in infection assays. *P. syringae* ice nucleating protein (INP) preparations were used as controls. **(B)** Representative non‐acclimated (NA) and cold‐acclimated (CA) whole plants sprayed with C.A. *P. syrinage* pathovar cultures and then maintained at 24 °C (two left pots) and −3 °C (two right pots). **(C)** Leaves taken from NA or CA plants with the cut ends dipped in cold‐activated *P. syringae* cultures, with the excised leaves then maintained at 24 °C (left pair of images) and −3 °C (right pair of images).

**Table S1** *Cis‐*acting regulatory elements of putative promoters for *Bd*IRI1–7 in *Brachypodium* and *Os*MYB1R35 from *Oryza sativa* as determined by PlantCARE.

**Table S2** Predicted miRNA binding of *BdIRI1–7* and method of inhibition as determined by psRNATarget miRNA prediction tool with data from miRBase (Release 21, June 2014).

## Data Availability

All data generated or analyzed during this study are included in this published article (and its supplementary information files).
