# Supplementary material for: Cold‐inducible promoter‐driven knockdown of Brachypodium antifreeze proteins confers freezing and phytopathogen susceptibility
Source: Plant Direct. 2022 Sep 12;6(9):e449. doi: 10.1002/pld3.449 (PMC9467863; doi:10.1002/pld3.449)
Supplement: Supplementary file 1 — Figure S1. Alignments performed using Clustal Omega (https://www.ebi.ac.uk/Tools/msa/clustalo/) of all 7 BdIRI amino acid sequences from the newest assembly ( Brachypodium distachyon genome v3) except that a likely frameshift mutation for BdIRI2, which appears in the reference genome but not in other accessions, is not shown here, and neither is the 22 amino acid extension on the N‐terminal in BdIRI4, which is likely a result of an additional or incorrectly noted start codon. The annotated apoplast localization signal sequence is shown in red, LRR motifs of LxxL where x represents a non‐conserved residue in green, and AFP motifs of NxVxG/NxVxxG where x represents an outward‐facing residue of the beta‐barrel structure in blue, along with the putative asparagine endopeptidase hydrolytic cleavage sites indicated by black arrows. Asterisks (*) denote fully conserved residues, colons (:) denote conservative substitutions, and periods (.) denote semi‐conservative substitutions. Figure S2. Illustration showing the three clusters and chromosomal positions of the 7 BdIRI genes on chromosome five of Brachypodium ( Brachypodium distachyon genome v3). BdIRI gene numbers are labeled and strands (+/−) are labeled and highlighted. Flanking and other nearby genes are shown with BdIRI genes are highlighted in blue. NCBI Entrez Gene IDs are labeled. The dotted line indicates the continuity of the chromosome. Figure S3. Representative western blot analysis for characterization of the rice promoter prOsMYB1R35 in Brachypodium distachyon using extracts from non‐acclimated (NA) and cold‐acclimated (CA) Bd21 wild type and prOeGFP transgenic plants and visualized with antibodies for green fluorescent protein (GFP). (A) Lane 1 corresponds to NA prOeGFP, lanes 2–5 to CA prOeGFP from separate individual plants, lane 6 to purified recombinant GFP used as a positive control, lane 7 to NA wild type, and lane 8 to CA wild type. (B) RuBisCO large chain (Rbcl) was used as a loading control with Coo [file PLD3-6-e449-s002.pdf]

```
BdIRI1          MAKCWLPHLLLA--LLLP-AASMAASCHPDDLRALRGFAGNLSGG-AVLLRATWSGASCC     56
BdIRI7          MAKCWLLHLLLA--LLLP-AASTAASCHPDDLHALRGFAGNLSGG-AVLLRATWFGASCC     56
BdIRI5          MAKCWLLMQLLL-ALLLPAAISMAAACHPHDLHALQGFAGNLSGG-AVLLRATWTGASCC     58
BdIRI6          MAKCWLLLQLLS-ALLLPAAISTAAACHPDDLRALRGFAGNLSGG-AVLLRATWSGASCC     58
BdIRI4          MAKCWLMQL-ILLTFLLPAAISTAVACHPDDLRVLRGFAGNLSGAGAVLLRATWSGASCC     59
BdIRI2          MAKYWLLLQLLSTLLILPAATSTAAACHPDDLRALRGFAGNLSGGAAVLLRAMWSGASCC     60
BdIRI3          MAKYWLLLQLLSALLILPAATSTAASCHPDDLRALRGFAGNLSGGAAVLLRAAWSGASCC     60
                *** **    :   ::** * * *.:***.**:.*:********. ****** * *****

BdIRI1          GWEGVGCDSA--SGRVTSLWLPGRGLTGPIQGAASLAGLVRLESLNLADNRLV--GTIPS     112
BdIRI7          GWEGVGCDGA--SGRVTTLWLPGRGLAGPIQGAASLAGLAQLESLNLANNRLV--GTIPS     112
BdIRI5          GWEGVGCNGGKSDGRVTTLLLPGRGLTGPILGASSLAGLAQLESLNLAHNRLQVGSTFPL     118
BdIRI6          GWEGVGCDGD--AGRVTTLSLPERGLAGPIQGAAPLAGLRQLESLNLAHNRLQVGTTFPS     116
BdIRI4          SWEGVGCNGGA-SGRVMTLWLPGRGLAGPIQGAASLAGLARLESLNLANNRLQVGTTFPS     118
BdIRI2          GWEGVSCDGGA-SGRVTTLWLPGRGLVGPIQGVSSLAGLAQLESLNLANNRLQIGTTFPS     119
BdIRI3          GWEGVRCDDGA-SGRVTTLWLPGRGLVGPIHGASSLAGLAQLESLNLANNRLHVGTTFPS     119
                .**** *:.    *** :* ** ***.*** *.: **** :*******.***    *:*

                                                          ↓
BdIRI1          WIGELDRLCYLDLSHNASVYEVAKINPSQRSRGVTVSTNRKTLDEEPNTITGTNNHVRSG     172
BdIRI7          WIGELDRLCYLDLSHNASVDEVHKINPSQRSLGVAVSTNRRTLGGEPNTITGTNNHVRSG     172
BdIRI5          WIGELGHLRYLDLSHNASPLHVNSS------------NRRTLAEGQPNTISGTNNSVRSG     166
BdIRI6          WIAEFDRLCYLDLSHNVSPLHVKSS------------SRRTLATGQPNTITGANNSVRSG     164
BdIRI4          WIGELDRLRYFDLSHNASSFHVKHG------------DRRTLADGQPNTITGTNNSVRSG     166
BdIRI2          WIGELDRLCYLDLSHNASPLHVKR-------------NRRNTS-----------------     149
BdIRI3          WIGELDRLCYLDLSHNASPLHVKR-------------NRRTLADGKPNTITGTNNSVKSG     166
                **.*:.:* *:*****.*  .*                .*  ↑

BdIRI1          KDNALSGNDNTVISGDNNVVTGNHNKILSGSHNAVSGHMHVVSGTYHVVTGNNNAVTRSH     232
BdIRI7          KDNALSGSDNTVISGNNNVVTGNHNKVLSGSHNAVSGHMHVVSGTYHVVTGNNNAVTRSH     232
BdIRI5          SGNTVMGEDNIVISGDNNVVSGKQNT---------------VTGSDNVVSGSNNSV----     207
BdIRI6          SGNTIMGDGNT------------------------------ITGDNNVVSGNNNTV----     190
BdIRI4          NGNTVSGGDNTVISGNNNVLSGNNNTIISGSDNVVSGTNQVVSGTNHIVTGSNNTV----     222
BdIRI2          ------------------------------------------------------------     149
BdIRI3          NGNTISGNDNVVISGNNNVVSGNHNKVVSGSDNAVSGNMHVVSGTHHVVTGTNNTV----     222

BdIRI1          NTASGNHNIVSGHHNTVSGDHNTVSGSHNTVSGSNHIVTGNNKVVT--     278
BdIRI7          NTASGNHNIVSGHHNTVSGDHNTVSGSHNTVSGSNHIVTGNNKVVT--     278
BdIRI5          ------------------------SGSHNTVSGSNHVVSGDNKVVTGG     231
BdIRI6          ------------------------TGSNNTVSGSNHIVNGDNLVVTGG     214
BdIRI4          ------------------------SGNNNNVSGSNHIVSGSNKIVTGG     246
BdIRI2          ------------------------------------------------     149
BdIRI3          ------------------------SGSNNAVSGSNHIVSGSNKVVTGG     246
```

Figure S1. Alignments performed using Clustal Omega (https://www.ebi.ac.uk/Tools/msa/clustalo/) of all 7 *BdIRI* amino acid sequences from the newest assembly (*Brachypodium distachyon* genome v3) except that a likely frameshift mutation for *BdIRI2*, which appears in the reference genome but not in other accessions, is not shown here, and neither is the 22 amino acid extension on the N-terminal in BdIRI4, which is likely a result of an additional or incorrectly noted start codon. The annotated apoplast localization signal sequence is shown in red, LRR motifs of LxxL where x represents a non-conserved residue in green, and AFP motifs of NxVxG/NxVxxG where x represents an outward-facing residue of the beta-barrel structure in blue, along with the putative asparagine endopeptidase hydrolytic cleavage sites indicated by black arrows. Asterisks (*) denote fully conserved residues, colons (:) denote conservative substitutions, and periods (.) denote semi-conservative substitutions.

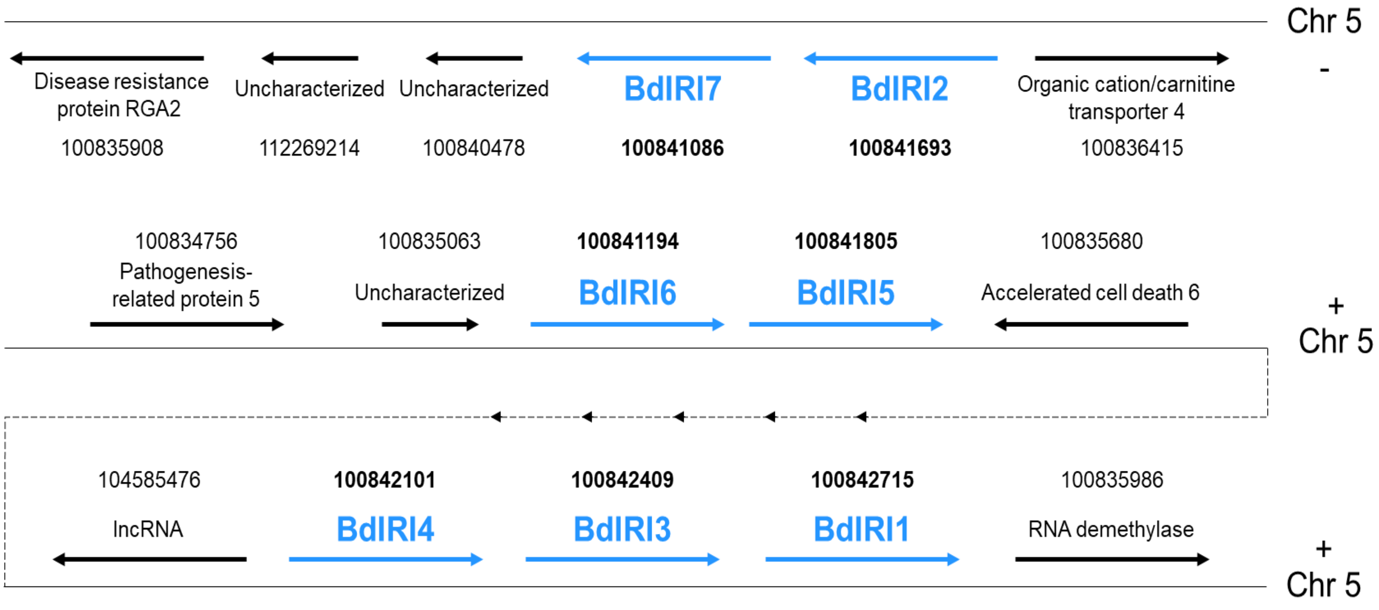

Figure S2. Illustration showing the three clusters and chromosomal positions of the 7 *BdIRI* genes on chromosome five of *Brachypodium* (*Brachypodium distachyon* genome v3). *BdIRI* gene numbers are labelled and strands (+/-) are labelled and highlighted. Flanking and other nearby genes are shown with *BdIRI* genes are highlighted in blue. NCBI Entrez Gene IDs are labelled. The dotted line indicates the continuity of the chromosome.

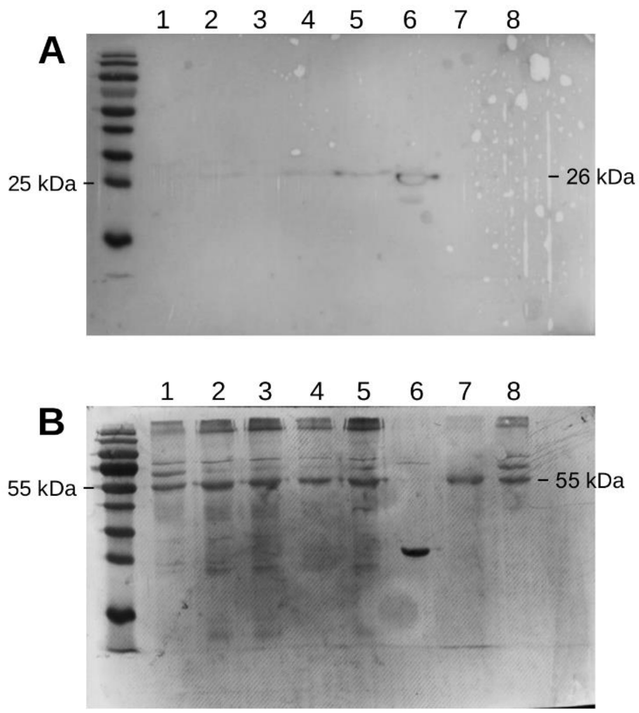

Figure S3. Representative western blot analysis for characterization of the rice promoter pr*OsMYB1R35* in *Brachypodium distachyon* using extracts from non-acclimated (NA) and cold-acclimated (CA) Bd21 wild type and prOeGFP transgenic plants and visualised with antibodies for green fluorescent protein (GFP). **(A)** Lane 1 corresponds to NA prOeGFP, lanes 2-5 to CA prOeGFP from separate individual plants, lane 6 to purified recombinant GFP used as a positive control, lane 7 to NA wild type, and lane 8 to CA wild type. **(B)** RuBisCO large chain (Rbcl) was used as a loading control with Coomassie Brilliant Blue staining. Molecular weights of bands corresponding to eGFP (26 kDa) and Rbcl (55 kDa), are labelled. Western blots were performed in triplicate. Note: positive control recombinant eGFP was overloaded and burnt during imaging.

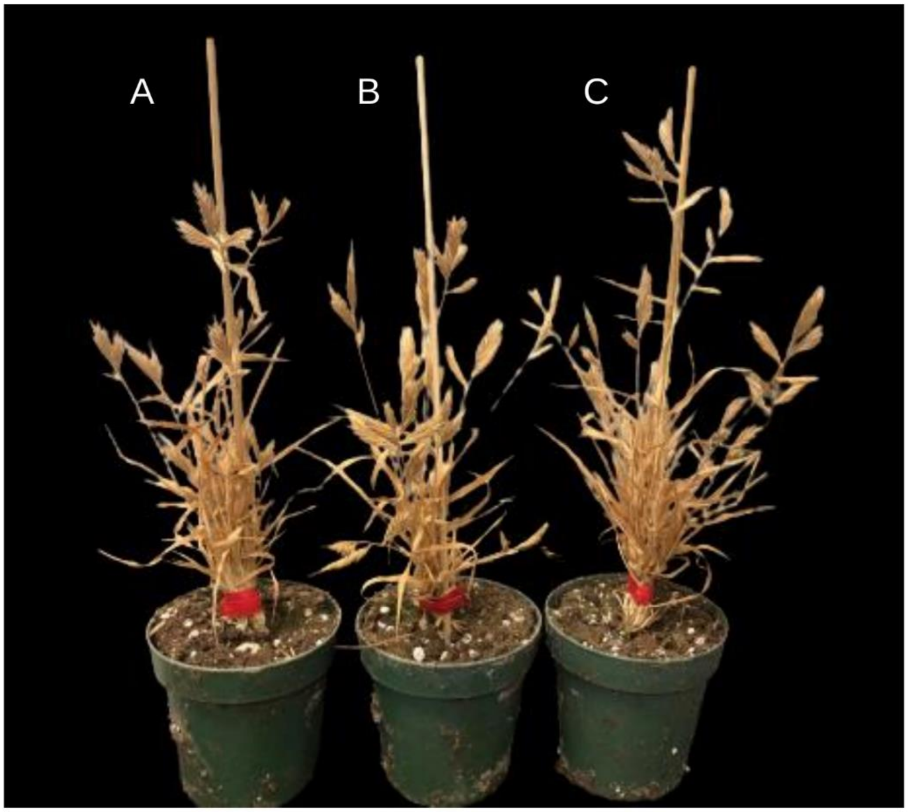

Figure S4. Phenotypes of senescent Bd21 wild type *Brachypodium distachyon* **(A)** plants and two homozygous knockdown lines, prOmiRBdIRI-1e **(B)** and prOmiRBdIRI-3c **(C)**, bearing our temporal AFP knockdown systems. Photos were taken at 12 weeks following the described standard growth conditions with water and fertilizer withdrawn in the final week to allow senesced plants to dry out for seed harvesting.

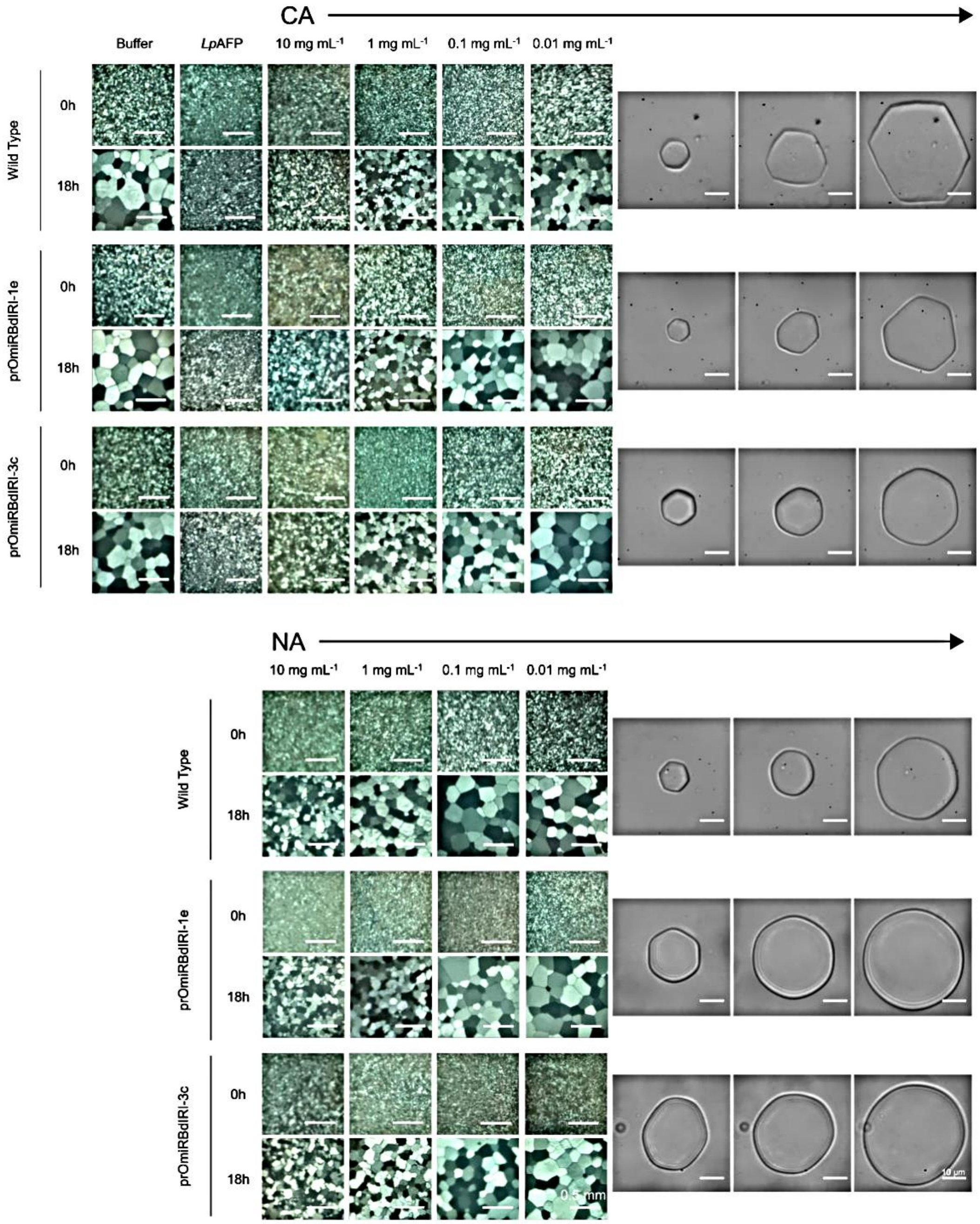

Figure S5. Ice recrystallization inhibition "splat" assays of cell protein extracts using leaf tissue from non-acclimated (NA) and cold-acclimated (CA) *Brachypodium distachyon* Bd21 wild type and temporal AFP knockdown lines prOmiRBdIRI-1e and prOmiRBdIRI-3c. Samples were annealed at -6 °C for 18 h at various concentrations. Buffer and *Lp*AFP controls are shown. Scale bar for splat assays represents 0.5 mm. Ice crystal morphologies and burst patterns are shown alongside and were tested at 40 mg mL^{-1} of total protein concentrated from crude cell extracts. Micrographs were captured at 50x zoom on a nanoliter osmometer with scale bars representing 10 µM. All assays were performed in triplicate with similar results and representative images are shown.

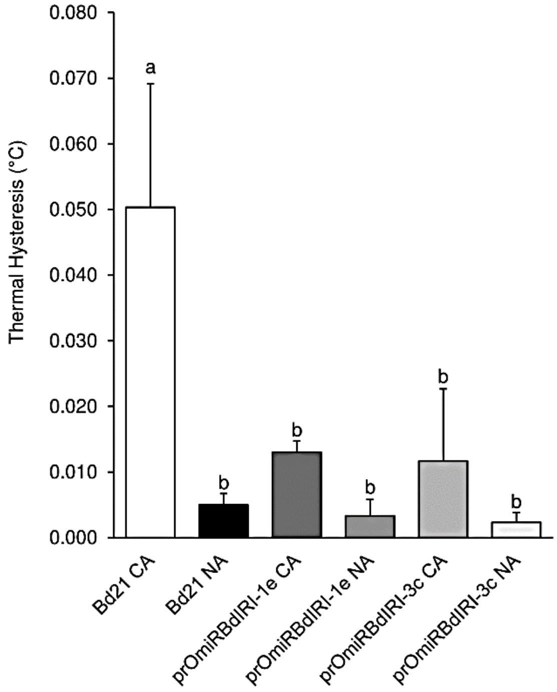

Figure S6. Thermal hysteresis (TH) readings for crude protein extracts from leaf tissue lysates on non-acclimated (NA) and cold-acclimated (CA) Bd21 wild type *Brachypodium distachyon* and two temporal AFP knockdown lines prOmiRBdIRI-1e and prOmiRBdIRI-3c. Samples were tested at 40 mg mL^{-1} of total protein concentrated from crude cell extracts. Readings were captured using a nanoliter osmometer and performed in triplicate. Letters represent statistically significant groups following one-way ANOVA with post-hoc Tukey multiple test correction ($p < 0.01$).

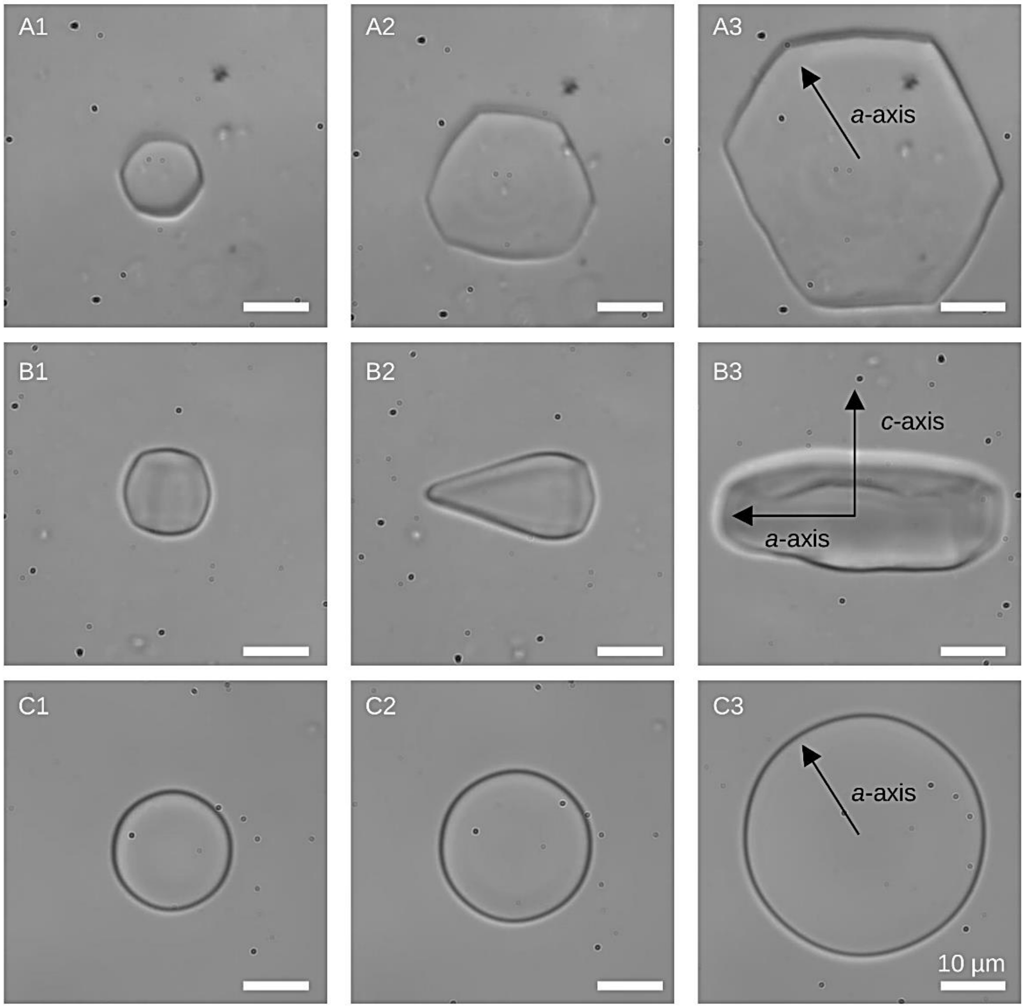

Figure S7. Ice crystal morphology and burst patterns in the presence of antifreeze proteins in crude lysates of wildtype cold-acclimated *Brachypodium distachyon* Bd21 leaf tissue viewed down on the *a*-axis **(A1-A3)** and viewed horizontally towards the *a*-axis **(B1-B3)**. *Brachypodium* AFPs have an affinity for the prism and basal planes. Buffer solution showing unrestricted ice crystal growth in the absence of AFPs with characteristic disk-shaped morphology visible, viewed down on the *a*-axis **(C1-C3)**. Micrographs were captured at 50x zoom on a nanoliter osmometer and performed in triplicate. Scale bar is 10 µm.

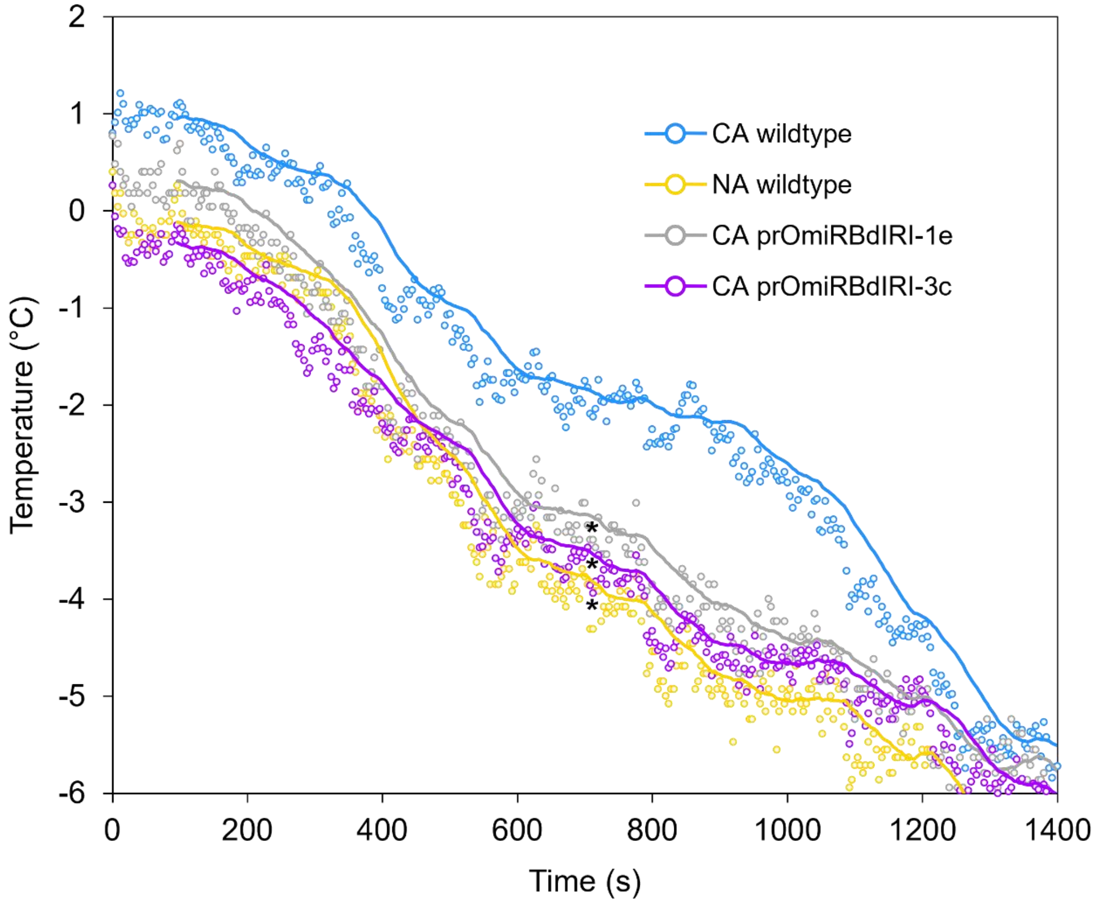

Figure S8. Infrared thermography assay performed on cold-acclimated (CA) wild type (blue), CA prOmiRBdIRI-1e (grey), CA prOmiRBdIRI-3c (purple) knockdown lines, and non-acclimated (NA) wild type (yellow). Data represent the recorded thermography data where each point represents the temperature at a single frame captured at a frame rate of 1 frame every 4 sec. Lines shown represent the moving averages using a period of 25. Points measured were 5 mm from the wounded end of the leaf where the tissue was excised from plants. Leaves were equilibrated at 1 °C for 30 min and frozen to -10 °C at a rate of 0.01 °C sec^{-1}. Experiments were performed in triplicate. CA prOmiRBdIRI-1e, CA prOmiRBdIRI-3c knockdown lines, and NA wild type were significantly different from CA wild type indicated by stars (one tailed *t*-test, unpaired).

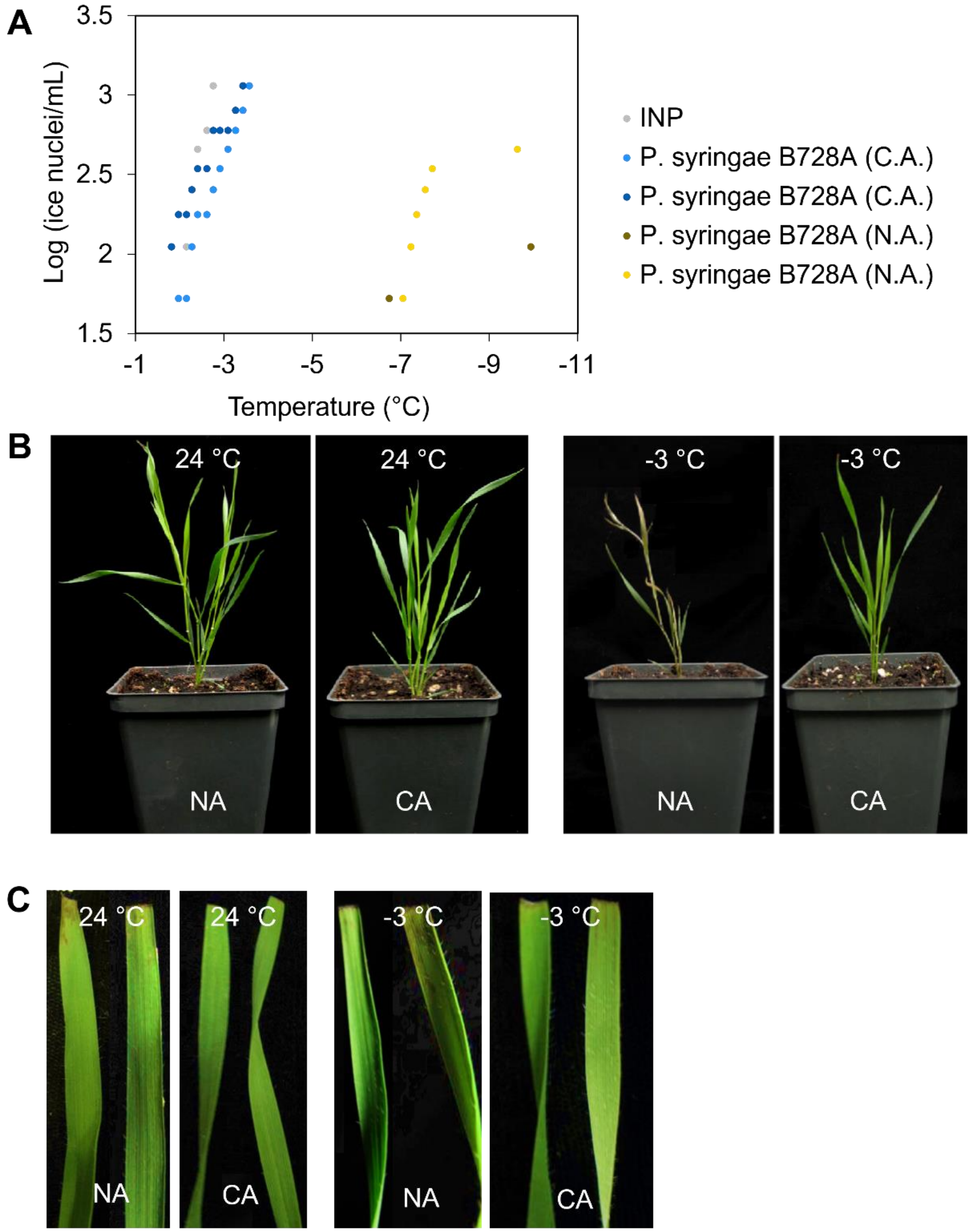

Figure S9. Ice nucleation activity and infection phenotypes for *Brachypodium distachyon* with *Pseudomonas syringae* pv. *syringae* B728A. **(A)** Ice nucleation activity of the *P. syringae* pathovar that had been cold-activated by transfer to 4 °C for 48 h (C.A.) or kept at 24 °C, representing non-cold-activated (N.A.) cultures with nucleation temperatures between -2 °C and -4 °C, were used in assays to determine the conditions and temperature of incubation for leaf tissue in infection assays. *P. syringae* ice nucleating protein (INP) preparations were used as controls. **(B)** Representative non-acclimated (NA) and cold-acclimated (CA) whole plants sprayed with C.A. *P. syrinage* pathovar cultures and then maintained at 24 °C (two left pots) and -3 °C (two right pots). **(C)** Leaves taken from NA or CA plants with the cut ends dipped in cold-activated *P. syringae* cultures, with the excised leaves then maintained at 24 °C (left pair of images) and -3 °C (right pair of images).
